# GL-Net: A knowledge-guided Gaussian-gated and layered refinement network for 3D MRI segmentation of brain gliomas

**DOI:** 10.1371/journal.pone.0351953

**Published:** 2026-06-22

**Authors:** Huimin Lu, Yilong Wang, Han Xue, Guizeng Wang, Jamshid Moradi Kurdestany, Songzhe Ma

**Affiliations:** 1 School of Mathematics and Statistics, Changchun University of Technology, Changchun, Jilin, China; 2 School of Computer Science and Engineering, Changchun University of Technology, Changchun, Jilin, China; 3 The First Hospital of Jilin University, Changchun, Jilin, China; 4 Department of Radiation Oncology, Mercy Hospital Cancer Center, Oklahoma, Oklahoma, United States of America; Hallym University, KOREA, REPUBLIC OF

## Abstract

Glioblastoma is a highly malignant brain tumor, and accurate lesion segmentation in MRI is essential for diagnosis, treatment planning, and prognosis assessment. This paper proposes a knowledge-guided 3D hybrid Transformer-CNN framework, GL-Net, which integrates prior knowledge through a Gaussian Gating Module (GGM) and a Layered Refinement Module (LRM), together with a novel Edge-Region Voxel Dynamic Weighted Loss Function. These modules collaboratively enhance feature activation, refine label-specific structures, and improve edge delineation, enabling robust segmentation even under limited-sample conditions. The proposed GL-Net was evaluated on the BraTS2019 and BraTS2021 datasets, achieving average Dice Similarity Coefficients (DSC) of 0.877 and 0.913, and Hausdorff Distances (HD) of 1.83 and 1.55, respectively—demonstrating highly competitive performance and a substantial reduction in boundary errors relative to the reported benchmarks of current data-driven approaches. Furthermore, to assess its clinical applicability, VASARI (Visually Accessible Rembrandt Images) feature extraction was performed using both the GL-Net-generated segmentation masks and the ground truth labels on the BraTS2019 dataset for glioblastoma (GBM) diagnosis. The diagnostic performances were nearly identical (GT AUC: 0.954 / GL-Net AUC: 0.949), and the DeLong test (p = 0.99) indicated no statistically significant difference between the two. These results suggest that GL-Net not only achieves highly competitive segmentation accuracy but also produces radiomic features comparable to expert manual annotations, providing complementary evidence of its potential clinical relevance. The proposed framework shows strong clinical potential for precise and consistent glioma delineation, providing valuable support for surgical planning, radiotherapy targeting, and diagnostic decision-making in clinical workflows.

## Introduction

Glioblastoma is a complex and severe neurological disease that profoundly affects patients’ lives and overall quality of life. In clinical practice, the characterization and treatment planning of gliomas largely rely on radiological interpretation based on standardized assessment systems. The VASARI (Visually Accessible Rembrandt Images) feature set [1,2] provides a structured vocabulary for describing glioma morphology, including enhancement patterns, necrosis, edema, and mass effect. Although these frameworks offer important guidance for clinical decision-making, they still depend heavily on expert visual assessment and manual delineation, which are time-consuming and subject to considerable inter-observer variability. With the rapid development of deep learning, medical imaging analysis has entered a new era [3]. Among these, CNN and Transformer architectures have gained considerable attention due to their exceptional feature-learning capabilities and ability to model complex relationships [4]. These models can learn from large volumes of medical imaging data, automatically extract features relevant to glioblastoma, and subsequently perform downstream computations [5,6], offering new perspectives for glioblastoma segmentation.

However, in practical semantic segmentation applications, the high heterogeneity and complexity of glioblastoma pose significant challenges. Models that rely solely on data-driven approaches often fail to achieve optimal performance, particularly in the following aspects: (1) The imaging characteristics of glioblastoma are highly diverse, with complex morphological and signal intensity variations on MRI. These factors make it difficult for algorithms based solely on image feature recognition to perform accurately; (2) Due to the relatively limited number of available cases, i.e., the small sample problem, training datasets are often insufficient to capture all potential patterns of disease variation. This frequently leads to overfitting and poor generalization in models [7], reducing accuracy in real-world predictions, especially when dealing with blurred tumor boundaries or overlapping regions.

To address these challenges, incorporating prior knowledge is critically important [8]. Prior knowledge may include information about the morphology and biological characteristics of glioblastoma, as well as domain expertise in the medical field [9]. By integrating such knowledge into deep learning models to enhance their regulatory constraints, the models can better understand the characteristics of glioblastoma, thereby improving the accuracy and robustness of segmentation results.

This study aims to develop a small-sample glioblastoma segmentation algorithm that seamlessly integrates deep learning with prior knowledge. To achieve this, the innovative GL-Net was designed. Through this algorithm, GL-Net enables more efficient recognition and segmentation of glioblastoma, providing more precise and reliable support for clinical diagnosis and treatment. Furthermore, we applied the model-generated segmentation results to the VASARI framework for GBM characterization and compared them with the gold-standard segmentations. This comparison highlights the potential clinical relevance and translational value of the proposed model. The main innovations of this study are as follows:

(1) GL-Net: By combining data-driven and knowledge-driven approaches, an innovative framework based on a hybrid structure of Transformer and CNN was designed. Prior knowledge is integrated into the model through the Gaussian Gating Module (GGM), Layered Refinement Module (LRM), and Edge-Region Voxel Dynamic Weighted Loss Function. This effectively leverages prior knowledge to enhance the overall performance of the model.
(2) Gaussian Gating Module: A secondary feature optimization module based on Gaussian functions was designed to effectively activate secondary features while suppressing overly sensitive or insensitive features. By leveraging the modulation capabilities of Gaussian functions, the module enhances the neural network’s ability to identify under-activated regions, such as the blurred edge areas of glioblastoma.
(3) Layered Refinement Module: A Layered Refinement Module based on label priors is proposed to progressively refine the prediction results for different label types, identifying and correcting mispredictions. This module employs a detail-aware extractor (DAE) that utilizes deep supervision and multi-scale feature fusion to extract and refine detailed features, thereby improving segmentation accuracy.
(4) Edge-Region Voxel Dynamic Weighted Loss Function: A novel loss function, DiceBRD loss, is introduced to focus more on the voxels in edge regions. Through a dynamic weighting mechanism, it alleviates the class imbalance problem. By adaptively adjusting the penalty for misclassified voxels in edge regions, this loss function enhances model accuracy in medical image segmentation tasks.
(5) The VASARI framework: The proposed GL-Net model is applied to the VASARI framework for GBM characterization, which is a comprehensive and reliable method for evaluating GBM. By comparing the model-generated segmentation results with the gold-standard segmentations, the potential clinical relevance of the proposed model is demonstrated.

The remaining sections of this paper are organized as follows: In the Related Work section, we introduce relevant studies on semantic segmentation algorithms that are purely data-driven. The Methods section details the experimental methods used in this study, including the overall framework of the model, the workflow, and the design details of the proposed innovative modules. The Experiment and Analysis section presents the main evaluation metrics, the datasets used, detailed experimental procedures, and a comparative analysis of the results.

## Related work

### The encoder-decoder structure of CNN models like U-Net

In previous studies, convolutional neural network (CNN)-based architectures have dominated the field of data-driven medical image semantic segmentation tasks, with models like U-Net [10], which adopt an encoder-decoder structure, has demonstrated particularly strong performance. The U-Net model achieves semantic segmentation of medical images through a symmetric encoder and decoder structure. The encoder extracts deep semantic features, progressively downsampling to capture global contextual information, while the decoder progressively upsampling to restore image resolution and reconstruct spatial details. Skip connections transfer high-resolution features between the encoder and decoder, ensuring that local details are preserved and improving the precision of edge segmentation. This model is particularly well-suited for small-sample medical image datasets, as it can capture global information while retaining local details.

In recent years, researchers have continuously improved and extended U-Net to address the challenges in the field of glioma segmentation. Some researchers have focused on introducing attention mechanisms to enhance segmentation performance by capturing significant regions. For example, Xu et al. [11] designed a novel U-Net by incorporating a corner attention module, which efficiently extracts dimensional information between slices and strengthens contextual connections, thus enhancing the network’s representational capacity. Schlemper et al. [12] proposed an Attention Gate (AG) model for medical image analysis, which highlights salient features useful for specific tasks. Akbar et al. [13] introduced a multi-path residual attention module based on the U-Net architecture, adding an attention gating mechanism in the skip connections to increase the model’s focus on target regions and reduce attention to non-target regions. Jia et al. [14] proposed an end-to-end glioma segmentation algorithm based on 3D U-Net, incorporating a coordinate attention module to enhance the ability to capture local texture features and global positional information. These improvements have significantly improved the network’s ability to accurately identify and segment regions of interest in glioma processing, providing strong support for glioma analysis.

### Transformer and its combined model with CNN

The Transformer [15] is a model architecture based on the Multi-Head Attention (MHA) mechanism. Through parallel processing and self-attention mechanisms, it can more effectively capture long-range dependencies in sequences. As a result, researchers have started using Transformer to explore its potential in extracting information across different modalities for glioma segmentation.

For example, Lin et al. [16] grouped input modalities into two categories based on MRI imaging principles and named the model CKD-TransBTS. This model leverages the Transformer’s advantages, capturing local lesion boundaries and extracting long-range features from 3D images. Hu et al. [17] proposed an efficient R-Transformer dual-encoder network, which captures complex semantic features and global contextual information by constructing a feature branch and a patch branch. Li et al. [18] introduced a new DenseTrans network that utilizes the shifted window operation of the Swin Transformer to obtain global feature information and long-range dependency modeling capabilities. Yang et al. [19] proposed a flexible multimodal glioma segmentation fusion network, which employs two Transformer-based feature learning networks and a cross-modal shared learning network to extract both individual and shared feature vectors, improving glioma segmentation performance using multimodal images. Ting et al. [20] introduced a multimodal Transformer to model correlations between multimodal features, progressively integrating multimodal and multilevel features for glioma segmentation using spatial and channel self-attention modules.

The aforementioned algorithms fully integrate the Transformer’s ability to capture global contextual information without being constrained by the receptive field, enabling better feature and structural information extraction in glioma image segmentation tasks. By employing methods such as the cross-fusion of global and local features, improvements to skip connection structures, and the integration of multimodal information, the accuracy and generalization capability of segmentation algorithms can be enhanced. While the self-attention mechanism in the Transformer captures global information well, it is less effective in handling local details. In images, pixels in local regions often exhibit strong correlations, and when processing images, the Transformer may not effectively capture this local correlation, leading to insufficient utilization of information.

Consequently, some researchers have developed hybrid architectures that integrate CNNs and Transformers. For example, Wang et al. [21] were the first to propose a novel glioma segmentation model called TransBTS, which combines CNNs and Transformers to extract spatial features and capture long-range dependencies. Liang et al. [22] proposed a TransConver model based on CNNs and Transformers, achieving cross-fusion of global and local features while improving skip connection structures to mitigate the semantic gap between encoder and decoder features for better fusion. Zhu et al. [23] introduced a glioma segmentation algorithm that utilizes multimodal MRI data and integrates semantic and edge information through deep learning techniques, aiming to fully leverage multimodal information.

In handling glioma segmentation tasks, these algorithms combine the advantages of classic deep learning models like CNNs, resulting in models with improved feature extraction and spatial information processing capabilities. By incorporating traditional algorithms, these models achieve a more comprehensive analysis and interpretation of medical images. However, the performance of CNN-Transformer architectures often relies on large amounts of annotated data. In the field of medical imaging, obtaining large quantities of annotated data can be challenging. Nevertheless, algorithms that incorporate prior knowledge can effectively address this issue. By leveraging the expertise and experience of medical professionals, models can be guided during training to better understand and analyze images, thus improving segmentation accuracy.

### Loss functions and the issue of class imbalance

In medical image analysis, tumor regions are significantly smaller than normal regions. Even within a single brain slice, the tumor area is much smaller than other parts of the brain, leading to class imbalance in medical images [24]. To effectively address this issue, researchers have worked on improving loss functions. Lin et al. [25] first proposed the focal loss for object detection in image segmentation, which introduces a modulation factor to alleviate the class imbalance problem. Caliva et al. [26] used a distance map as the weight for cross-entropy, allowing the loss to focus on difficult-to-segment boundary regions. Kervadec et al. [27] developed an edge loss algorithm that calculates region interfaces by integrating along boundaries, formulating it as a distance metric in contour space. Karimi et al. [28] introduced a loss function based on the Hausdorff Distance (HD) to directly minimize the HD between the model-generated contours and the ground truth. Liu et al. [24] proposed a multi-level structural loss by utilizing region, boundary, and pixel information to supervise feature fusion and achieve precise segmentation. Yeung et al. [29] proposed a Unified Focal Loss to address the issues of excessive hyperparameters and overly fast convergence in general focal loss. Du et al. [30] developed a boundary-sensitive loss function that automatically focuses on hard-to-segment boundaries, leading to more refined target delineation.

Although these algorithms help mitigate class imbalance to some extent, they still have limitations. For example, some algorithms rely on manually tuned hyperparameters or complex computational processes, increasing the difficulty of optimization. Additionally, certain methods do not sufficiently emphasize boundary regions, which can limit segmentation precision.

## Materials and methods

### Materials

The glioma MRI data used in this study were obtained from the standardized multi-center TCGA and TCIA repositories [33–35]. The BraTS2019 training set includes 259 high-grade glioma (HGG) and 76 low-grade glioma (LGG) cases, while the BraTS2021 dataset comprises 1,251 cases in total. The scans were collected from 13 independent medical institutions using MRI systems from GE, Siemens, and Philips, covering a magnetic field strength range of 0.5 T-3 T and incorporating diverse acquisition protocols (slice thickness: 1–3 mm, TR: 500–3000 ms, TE: 10–100 ms, matrix size: 128×128–512×512). This inherent clinical and technical heterogeneity enables GL-Net to learn tumor representations that are independent of specific scanners and acquisition protocols, thereby supporting its robust generalization under real-world imaging conditions.

### Methods

**Fig 1:**
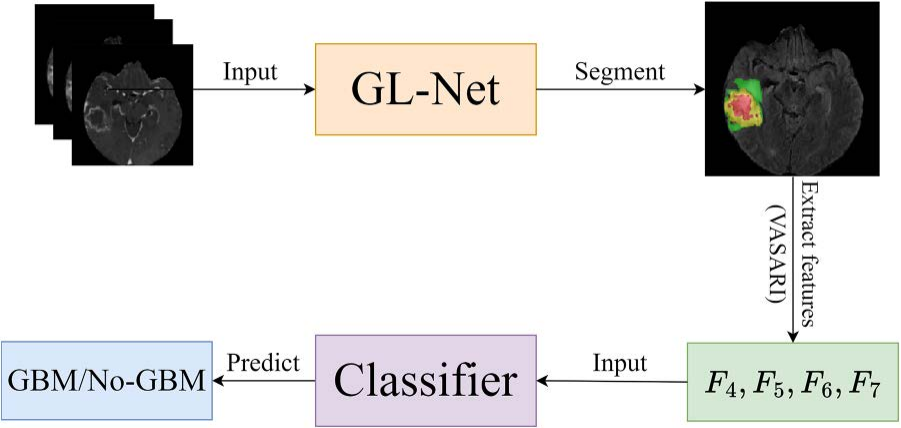
Clinical integration workflow of GL-Net segmentation.

**Fig 2:**
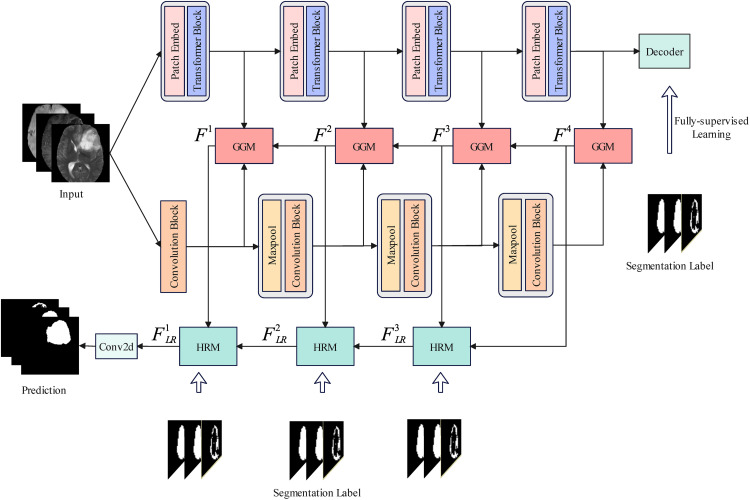
Network architecture of GL-Net.

Compared to natural images, extracting feature information from medical images is significantly more challenging, especially in multi-sequence medical imaging. Since optimal tumor image slices cannot always be guaranteed, our overall model strategy is built on 3D data to better adapt to practical applications (Fig 1). In this study, we designed a novel Gaussian-gated and secondary refinement segmentation algorithm, GL-Net, based on T-CNN (as shown in Fig 2). To fully integrate layered feature information from different structures, we introduce a Gaussian-gated module for secondary feature optimization, effectively fusing feature representations from both sides. On the decoder side, we design a label-prior-based LRM, which progressively refines the WT, TC, and ET regions, ultimately achieving accurate segmentation. Additionally, we propose a new region-dynamic-weighted loss function that mitigates the class imbalance problem by adjusting the computation domain and enhancing the decoder’s focus on image edges.

#### Gaussian gating module for secondary feature optimization.

**Fig 3:**
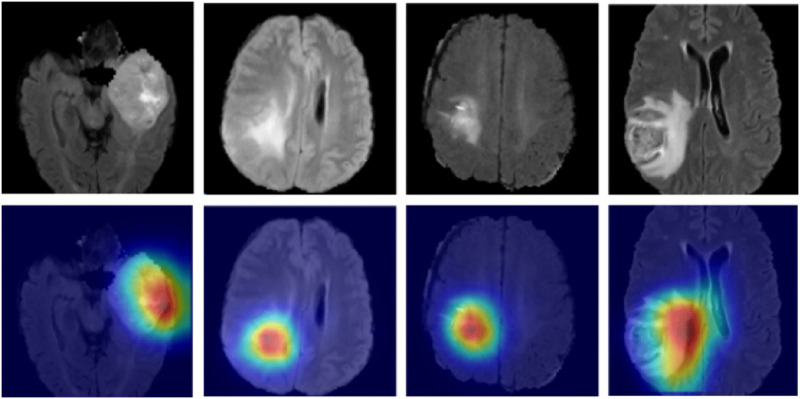
Activation mapping of glioma region.

**Fig 4:**
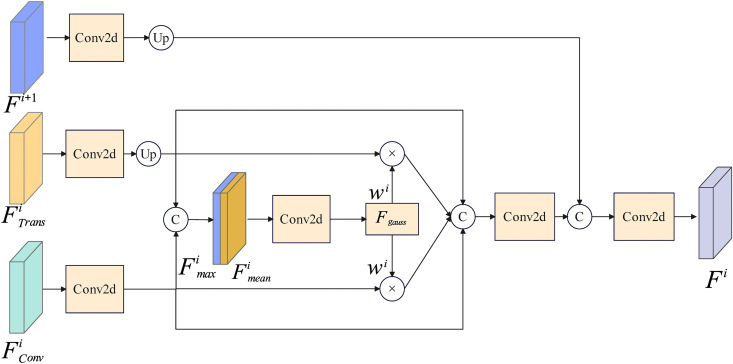
Gaussian gating module for secondary feature optimization.

To effectively utilize the unique features of T-CNN, it is essential to address the issues of noise in layered features and the compatibility of intrinsic differences. To achieve this goal, this paper introduces a Gaussian Gating Module for secondary feature optimization. The Gaussian function enhances secondary features while suppressing both the most and least sensitive features [31]. In deep learning-based medical image segmentation, secondary features refer to regions that are not fully activated during the neural network’s learning process. As shown in Fig 3, these unactivated regions are primarily distributed along the fuzzy boundary areas of gliomas. To effectively activate secondary features, this paper proposes a Gaussian Gating Module for secondary feature optimization leveraging the modulation properties of Gaussian functions, as illustrated in Fig 4.

First, the feature vectors $F_{Trans}^{i}$ extracted by MHA and $F_{Conv}^{i}$ extracted by the convolution kernel are passed through a 31×13 convolution kernel to reduce the original number of channels to $C_{i}/2$, which helps alleviate the computational burden of subsequent operations. To ensure spatial alignment between $F_{Trans}^{i}$ and $F_{Conv}^{i}$, $F_{Trans}^{i}$ needs to undergo an upsampling operation. Next, the two feature maps are merged through a concatenation operation, followed by average pooling (AvgPool) and max pooling (MaxPool) operations to obtain the feature vectors $F_{Trans}^{i}$ and $F_{Conv}^{i}$, respectively. Then, the output is passed through a 31×13 convolutional layer, and the resulting feature map is further processed using a Gaussian function to obtain the normalized weight $w^{i}\in [0,1]$. This weight reactivates the secondary features of both, compensating for the lost edge features. Finally, $F_{Trans}^{i}$ and $F_{Conv}^{i}$ are multiplied by the normalized weight $w^{i}$. The above process can be expressed by Equations (1) to (5):


$$\begin{array}{c} F_{mean}^{i}=mean(\{Up(Conv(F_{Trans}^{i})),Conv(F_{Conv}^{i})\}) \end{array}$$ (1)

$$\begin{array}{c} F_{max}^{i}=\max(\{Up(Conv(F_{Trans}^{i})),Conv(F_{Conv}^{i})\}) \end{array}$$ (2)

$$\begin{array}{c} w^{i}=gauss(Conv(\{F_{mean}^{i},F_{max}^{i}\})) \end{array}$$ (3)

$$\begin{array}{c} F_{Trans}^{i^{\prime }}=w^{i}⊗F_{Trans}^{i} \end{array}$$ (4)

$$\begin{array}{c} F_{Conv}^{i^{\prime }}=w^{i}⊗F_{Conv}^{i} \end{array}$$ (5)

Next, the activated feature maps are concatenated. To preserve the original information of the feature representations $F_{Trans}^{i}$ and $F_{Conv}^{i}$, the activated features are concatenated again with $F_{Trans}^{i}$ and $F_{Conv}^{i}$, followed by a convolution operation to generate the feature representation $F^{\left(i^{\prime }\right)}$. The above process can be expressed by Equation (6):


$$\begin{array}{c} F^{\left(i^{\prime }\right)}=Conv(\{F_{Trans}^{\left(i^{\prime }\right)},F_{Conv}^{\left(i^{\prime }\right)},Up(Conv(F_{Trans}^{i})),Conv(F_{Conv}^{i})\}) \end{array}$$ (6)

In addition, to retain the contextual information from the previous layer in the encoder, the fused feature $F^{\left(i^{\prime }\right)}$ can be combined with the output $F^{i+1}$ of the Gaussian Gating Module from the previous layer through a concatenation operation. Finally, the features of both are extracted through a convolutional layer. This process can be expressed by Equation (7):


$$\begin{array}{c} F^{i}=Conv(\{F^{\left(i^{\prime }\right)},Up(Conv(F^{\left(i+1\right)}))\}) \end{array}$$ (7)

In the equation, $F^{i}$ and $F^{i+1}$ represent the outputs of the Gaussian Gating Module at the current level and the previous level, respectively.

#### Layered refinement module based on label priors.

**Fig 5:**
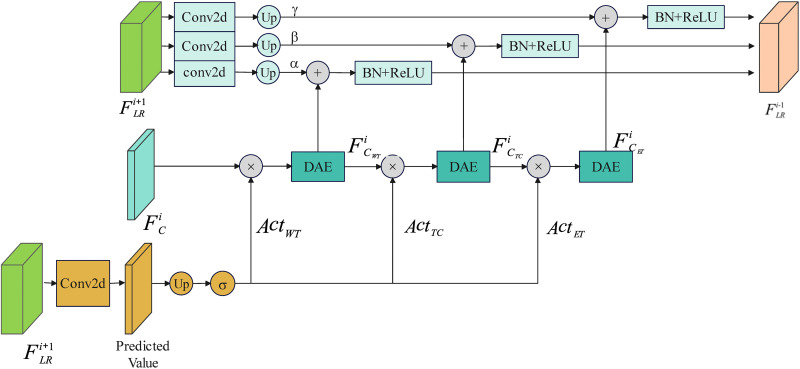
LRM based on label prior.

To address the impact of unclear boundaries and overlapping regions, this paper introduces an LRM. Inspired by the labeling format of gliomas, it is designed to progressively refine the prediction results for different types of labels, as shown in Fig 5. The purpose of this module is to identify and correct mispredictions. It takes deep features and current layer features as input and outputs the refined features along with the current layer’s prediction results.

First, the deep features $F_{LR}^{\left(i+1\right)}=\{F_{WT}^{\left(i+1\right)},F_{TC}^{\left(i+1\right)},F_{ET}^{\left(i+1\right)}\}$, $F_{LR}^{\left(i+1\right)}\in {\mathbb{R}}^{3\times N\times C\times H\times W}$, and their components $F_{WT}^{\left(i+1\right)},F_{TC}^{\left(i+1\right)},F_{ET}^{\left(i+1\right)}$ undergo convolution operations to reduce the number of channels to 1, resulting in the corresponding predicted maps. Second, deep supervision is applied to the generated predicted maps, guiding the detail-aware extraction module to refine the detail features of the corresponding predictions. Finally, upsampling and activation operations are performed sequentially to obtain the corresponding activation values. The specific process is described in Equation (8):


$$\begin{array}{c} Act_{j}=\sigma (Up(Conv(F_{LR_{j}}^{\left(i+1\right)}))) \end{array}$$ (8)

In the formula, $F_{LR}^{\left(i+1\right)}$ represents the deep features of the (*i* + 1)-th layer, ${Act}_{j}$ denotes the activation value for refining the *j*-th target region, where *j* takes values from {*WT*, *TC*, *ET*}. σ represents the Sigmoid function.

Secondly, this paper multiplies ${Act}_{WT}\in {Act}_{j}$ with the current feature $F_{C}^{i}$ to generate the foreground attention feature for *WT*. Subsequently, this feature is fed into the DAE to perform the detail extraction task, resulting in the detail feature $F_{C_{WT}}^{i}$ for *WT*. Then, ${Act}_{TC}$ is multiplied with $F_{C_{WT}}^{i}$ to obtain the foreground attention feature for *TC*, and the detail feature $F_{C_{TC}}^{i}$ for *TC* is extracted using the DAE. Similarly, the detail feature $F_{C_{ET}}^{i}$ can be obtained. This process can be expressed by Equation (9).


$$\begin{array}{c} F_{C_{j}}^{i}=\text{DAE}({Act}_{j}⊙F_{C_{\left(j-1\right)}}^{i}) \end{array}$$ (9)

In the formula, $F_{C_{\left(j-1\right)}}^{i}$ represents the detail feature from the previous layer of the DAE for $F_{C_{j}}^{i}$, where *j* takes values sequentially from {*WT*, *TC*, *ET*}; *j* denotes the DAE.

Then, this paper first performs convolution and upsampling operations on the deep feature $F_{LR}^{\left(i+1\right)}$, and then adds it to the features extracted by the DAE. Secondly, normalization and nonlinear activation are performed through the BR layer on $F_{LR}^{\left(i-1\right)}$. This process can be expressed by Equations (10) to (14).


$$\begin{array}{c} F_{C_{j}}^{i}=\text{DAE}({Act}_{j}⊙F_{C_{\left(j-1\right)}}^{i}) \end{array}$$ (10)

$$\begin{array}{c} F_{WT}^{\left(i-1\right)}=BR(F_{C_{WT}}^{i}+\alpha F_{f_{WT}}^{i}) \end{array}$$ (11)

$$\begin{array}{c} F_{TC}^{\left(i-1\right)}=BR(F_{C_{TC}}^{i}+\beta F_{f_{TC}}^{i}) \end{array}$$ (12)

$$\begin{array}{c} F_{ET}^{\left(i-1\right)}=BR(F_{C_{ET}}^{i}+\gamma F_{f_{ET}}^{i}) \end{array}$$ (13)

$$\begin{array}{c} F_{LR}^{\left(i-1\right)}=\{F_{WT}^{\left(i-1\right)},F_{TC}^{\left(i-1\right)},F_{ET}^{\left(i-1\right)}\} \end{array}$$ (14)

In the formula, BR(·) denotes the BN + ReLU operation.

**Fig 6:**
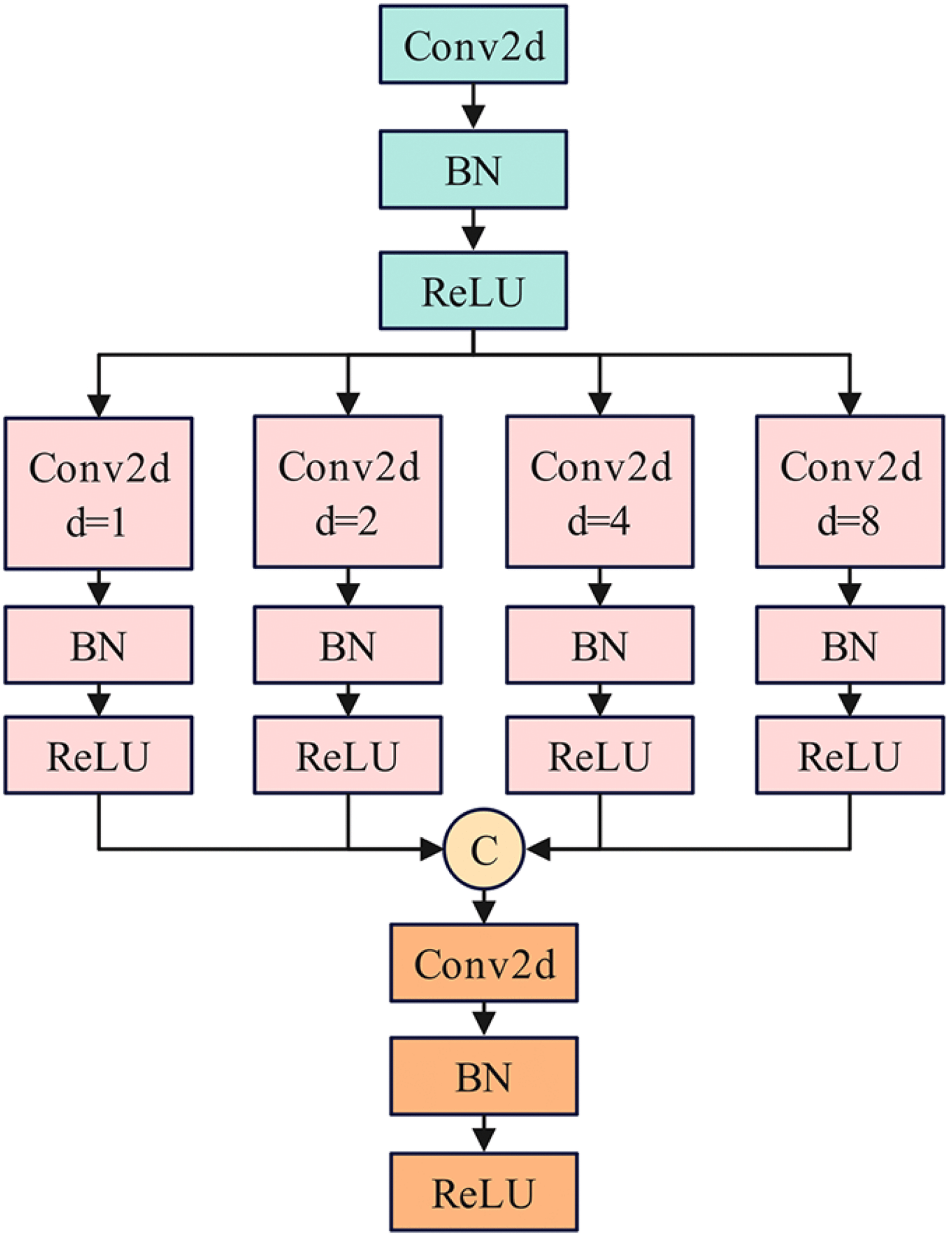
Structure of the detail-aware extractor for multi-scale feature fusion.

**Multi-scale feature fusion DAE:** The input features are first processed through a 1×1 convolution kernel, reducing the original channel size $C_{i}$ to $C_{i}/4$, which helps reduce the computational burden of subsequent operations. This is followed by a batch normalization layer and a ReLU operation. Four parallel branches are then established to provide various receptive fields for matching candidate regions of different sizes and shapes. In each branch, a 3×3 dilated convolution kernel is used, with a specific dilation rate and padding size. These four branches are then merged to obtain the fused features. Finally, a 1×1 convolution kernel, batch normalization layer, and ReLU operation are applied to fuse the concatenated features, resulting in the detailed feature vector, as shown in Fig 6.

#### Dynamic weighted loss function for edge region voxels.

**Fig 7:**
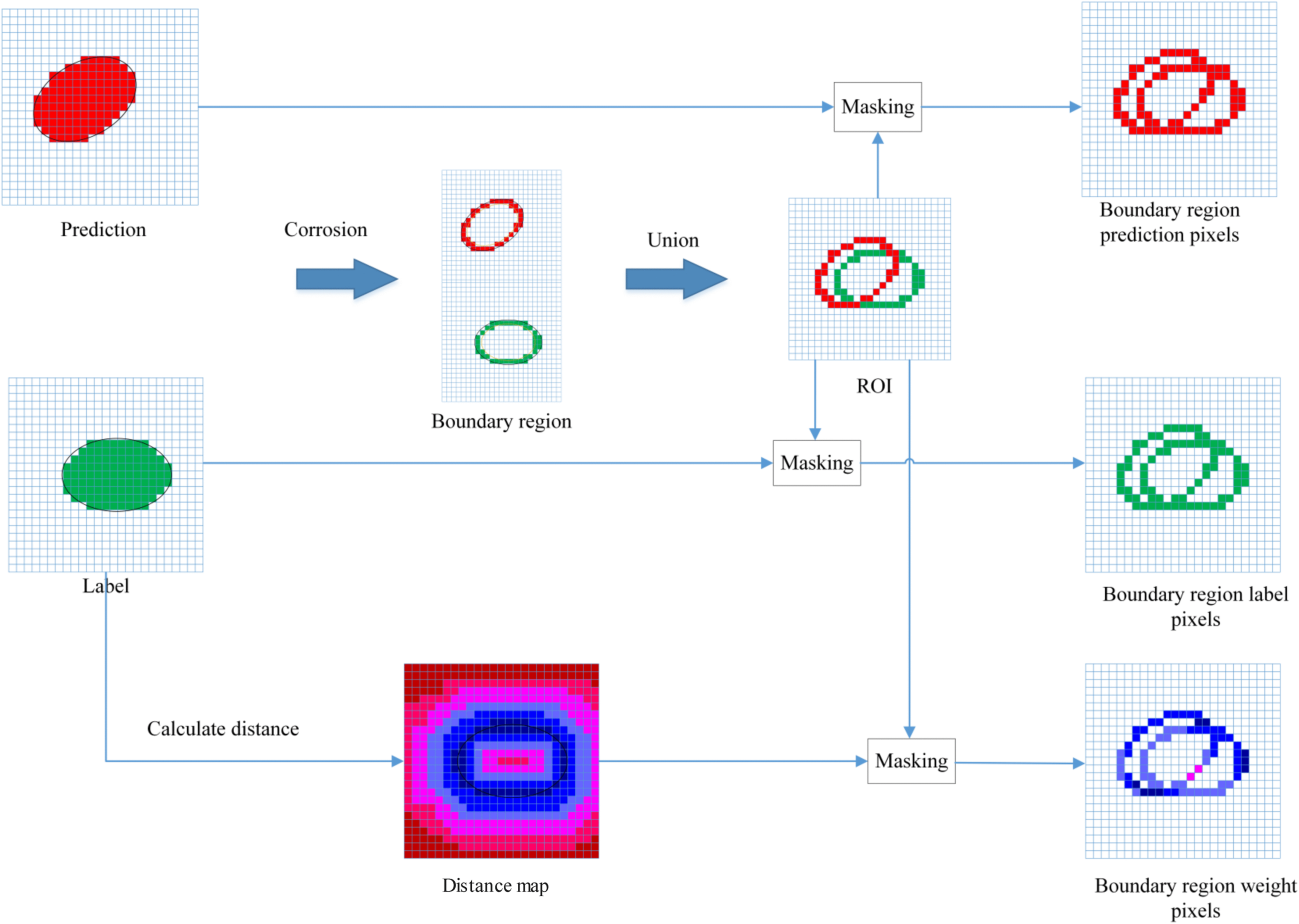
Flowchart of the dynamic weighted loss function for edge region voxels. Red, green, and white areas represent the predicted values, ground truth labels, and background, respectively.

In medical images, the presence of abundant noise and artifacts often leads to blurred target edge contours, which significantly affects the loss function. To address this issue, this paper introduces an edge-region voxel dynamic weighting loss function, called DiceBRD loss [32], as shown in Fig 7. This loss not only alleviates the class imbalance problem caused by the asymmetry between the number of foreground and background voxels but also precisely identifies the true edges by adaptively adjusting the penalty for misclassified voxels in edge regions. This improves the accuracy of deep neural networks in medical image segmentation.

DiceBRD loss extracts the edges of both the predicted results and the ground truth labels during the training iterations as the regions for loss calculation. The union of these regions defines the region of interest (ROI) for recalculating the loss. The edge extraction process can be expressed using equations (15) to (17).


$$\begin{array}{c} R_{\text{logits}}=I_{\text{SourceLogits}}-I_{\text{ErosionLogits}} \end{array}$$ (15)

$$\begin{array}{c} R_{\text{targets}}=I_{\text{SourceTargets}}-I_{\text{ErosionTargets}} \end{array}$$ (16)

$$\begin{array}{c} R=R_{\text{logits}}\cup R_{\text{targets}} \end{array}$$ (17)

Here, *R* represents the dynamically extracted boundary region of interest (ROI). The overall loss function, *L*_DiceBRD_, is formulated as a combination of a global Dice loss (*L*_Dice_) calculated over the entire image volume, and a Boundary Region-restricted Dynamic weighted cross-entropy loss (*L*_BRD_) calculated strictly within the dynamic ROI *R*.

To integrate both global topological structure and local boundary refinement, the proposed *L*_DiceBRD_ elegantly decouples the learning objectives. The overall loss is formulated as a weighted sum of the global Dice loss (*L*_Dice_) calculated over the entire image volume, and the Boundary Region-restricted Dynamic weighted cross-entropy loss (*L*_BRD_) calculated strictly within the dynamically extracted boundary region.

Specifically, the global *L*_Dice_ is defined as Equation (18):


$$\begin{array}{c} L_{\text{Dice}}=1-\frac{2\sum_{i\in V}\sum_{c=0}^{C}y_{ic}p_{ic}+\varepsilon }{\sum_{i\in V}\sum_{c=0}^{C}(y_{ic}+p_{ic})+\varepsilon } \end{array}$$ (18)

The local boundary-restricted term *L*_BRD_ is defined as Equation (19):


$$\begin{array}{c} L_{\text{BRD}}=-\frac{1}{\left|R\right|}\sum_{i\in R}\sum_{c=0}^{C}w_{i}y_{ic}\log(p_{ic}) \end{array}$$ (19)

Finally, the concise combined loss equation is explicitly formulated as Equation (20):


$$\begin{array}{c} L_{\text{DiceBRD}}=L_{\text{Dice}}+\lambda L_{\text{BRD}} \end{array}$$ (20)

To ensure absolute clarity, all symbols utilized in the above formulations are consistently defined as follows:

- *V*: The set of all voxels in the entire global image volume.
- *R*: The dynamically extracted boundary region of interest (R⊆V).
- |*R*|: The total number of voxels strictly within the boundary region *R*.
- *C*: The total number of segmentation classes (including the background).
- $y_{ic}\in \{0,1\}$: The ground truth one-hot encoding indicating whether voxel *i* belongs to class *c*.
- $p_{ic}\in [0,1]$: The predicted probability that voxel *i* belongs to class *c*.
- $w_{i}$: The dynamic adaptive weight, computed as the Euclidean distance from voxel *i* to the exact boundary, heavily penalizing boundary misclassifications.
- ε: A small smoothing factor added to prevent division by zero.
- λ: A hyperparameter balancing the contribution of the local boundary refinement against the global structural loss (in our implementation, λ is set to 1.0).

By defining the Dice term over the global domain *V*, the model effectively mitigates the severe class imbalance inherent in medical images. Conversely, by restricting the weighted cross-entropy term (*L*_BRD_) strictly to the dynamically evolving boundary domain *R* via the balancing weight λ, the model adaptively forces the network to refine blurry contours without overwhelming the loss gradients with easy background voxels.

#### GBM diagnosis based on VASARI.

In this study, we developed an automated pipeline for extracting VASARI features F4-F7 based on multi-sequence MRI and tumor segmentation masks. First, all MRI sequences were resampled to the spatial coordinate system of the label image to ensure voxel-wise spatial consistency. Tumor subregions—Whole Tumor (WT), tumor core (TC), and Enhancing Tumor (ET)—were defined according to the BraTS specifications. Additionally, connected component analysis was applied to remove small-volume noise and obtain stable tumor masks. The specific extraction rules are as follows:

F4 (Enhancement quality): Describes the degree of signal enhancement on post-contrast T1-weighted MRI, categorized as Absent, Minimal, or Avid. This requires comparing signal intensity changes in the tumor region between pre- and post-contrast T1-weighted images.
F5 (Enhancing Tumor proportion): The percentage of the tumor composed of enhancing regions.
F6 (Non-Enhancing Tumor proportion): The percentage of the tumor composed of non-enhancing regions.
F7 (Necrosis proportion): The percentage of the tumor composed of necrotic regions. These extracted features were subsequently used as inputs to a logistic regression classifier for GBM prediction.

## Results

### Dataset and evaluation metrics

This paper conducts experiments on the BraTS2019 and BraTS2021 [33–35] datasets to validate the effectiveness of the proposed algorithm. The BraTS2019 training set consists of 259 HGG and 76 LGG cases. The BraTS2021 official training dataset consists of 1,251 cases. Since the ground truth labels for the official validation and test sets of BraTS2019 and BraTS2021 are withheld by the challenge organizers and are not publicly available, all models in this study were trained and evaluated exclusively on the official training datasets. To ensure a robust and fair evaluation, a five-fold cross-validation strategy was performed. Therefore, any reference to “test set” or “test results” in this paper specifically denotes the internal hold-out test folds partitioned during this cross-validation process, rather than the official unseen BraTS test sets. The average results of the five runs are reported. In addition, for BraTS2019, we compared GBM predictions based on VASARI features extracted from the GL-Net-generated masks and from the gold-standard masks.

To evaluate the model’s potential clinical relevance, the following evaluation metrics(21–22) will be used:


$$\begin{array}{c} DSC=\frac{2TP}{2TP+FP+FN} \end{array}$$ (21)

$$\begin{array}{c} HD(x,y)=\max\left[\underset{x\in X}{\sup}\underset{y\in Y}{\inf}d(x,y),\underset{y\in Y}{\sup}\underset{x\in X}{\inf}d(x,y)\right] \end{array}$$ (22)

In this equation, *TP* represents the cases where the model correctly predicts the positive class. *FP* represents the instances where the model incorrectly predicts the negative class as positive. *FN* refers to the cases where the model incorrectly predicts the positive class as negative. *X* and *Y* are two proper subsets of the metric space *M*, with sup representing the supremum and inf representing the infimum.

For GBM diagnosis based on VASARI, commonly used classification metrics were employed, including ACC (Accuracy), Precision, Sensitivity (Recall), Specificity, F1-score, and AUC (Area Under the Curve).

Furthermore, to rigorously validate the performance improvements of the proposed GL-Net, statistical significance testing was conducted. The quantitative segmentation results across the five-fold cross-validation are reported as Mean ± Standard Deviation (SD). A paired Student’s t-test was utilized to determine whether the performance differences between the baseline configurations (or comparative models) and the proposed GL-Net were statistically significant across the test folds. A *p*-value of < 0.05 was considered statistically significant, and *p* < 0.01 was considered highly significant.

### Experimental setup and preprocessing

All experiments in this paper use MRI data provided by the BraTS2019 and BraTS2021 datasets. First, padding and cropping are performed to resize the images to 160 × 160 × 160, followed by Z-score normalization, where the mean of the normalized data is 0 and the variance is 1. Then, slice operations are carried out along the axial plane with a channel depth of 1, reducing the image size to 160 × 160. All experiments were conducted on an Ubuntu operating system equipped with an Intel(R) Xeon(R) CPU E5-2678 v3 @ 2.50GHz, 128 GB of system RAM, and dual NVIDIA RTX 3090 Ti GPUs (24 GB VRAM each). The proposed GL-Net has a total parameter count of approximately 35.2 M. Under this hardware configuration, the total training time for 100 epochs was approximately 14 hours, and the average inference time per patient volume during the testing phase was around 4.2 seconds, which fully meets the efficiency requirements for clinical practice.

For the training phase, the input image size is 3×160×160, the batch size is set to 8, and the number of epochs is 100. The optimization method used is SGD, with a weight decay of 0.0001, momentum set to 0.99, and the initial learning rate *lr*_0_ set to 1.5 × 10^−3^. The learning rate changes as the number of training iterations increases, as described by the following equation (22):


$$\begin{array}{c} lr=lr_{0}\times (1-\frac{\text{epoch}}{100}{)}^{0.9} \end{array}$$ (23)

Our code will be open-sourced at https://github.com/Turing17/GL-Net.

### Comparative experiments of GL-Net

**Fig 8:**
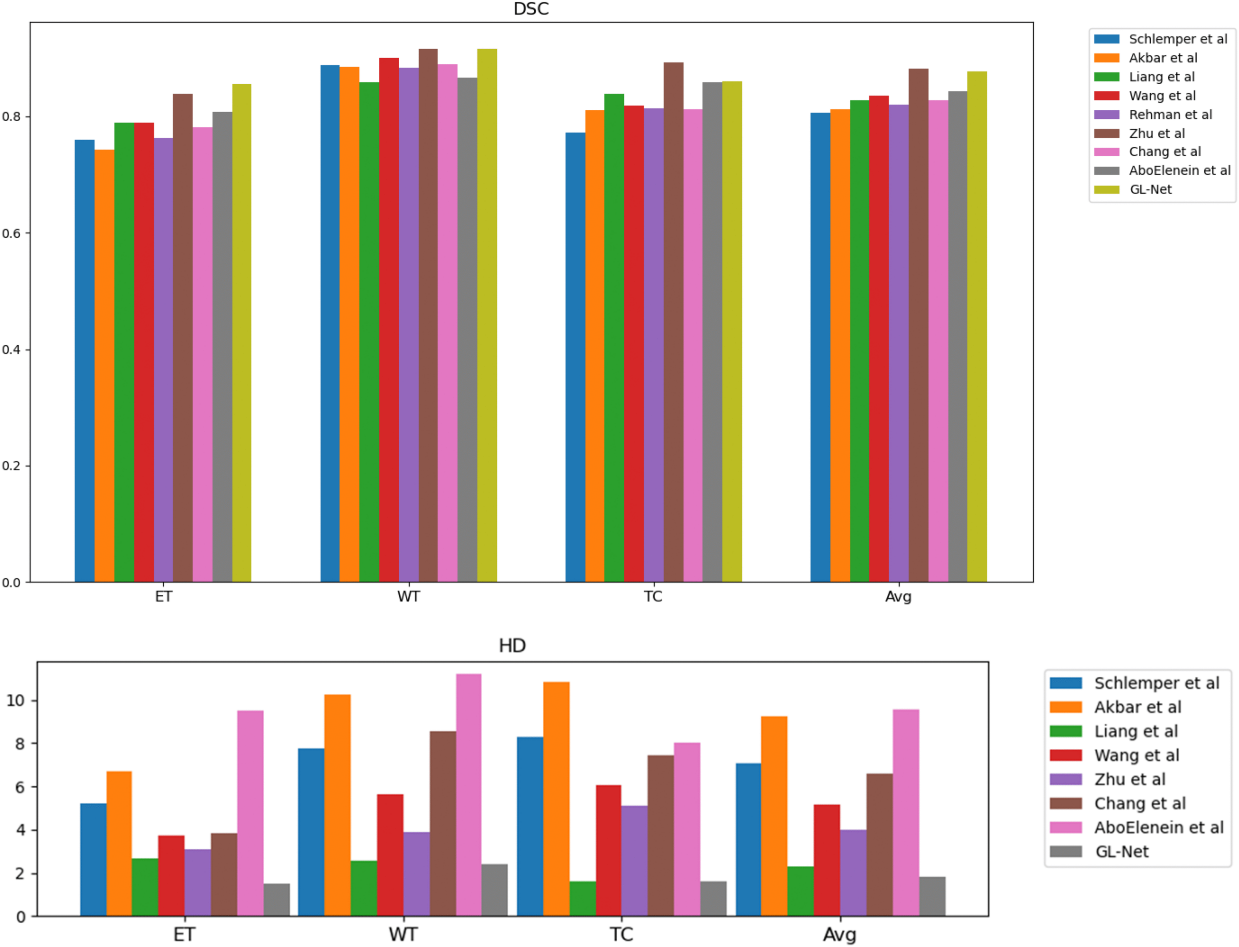
Bar chart of the comparison experiment results of GL-Net with other models on the BraTS2019.

**Fig 9:**
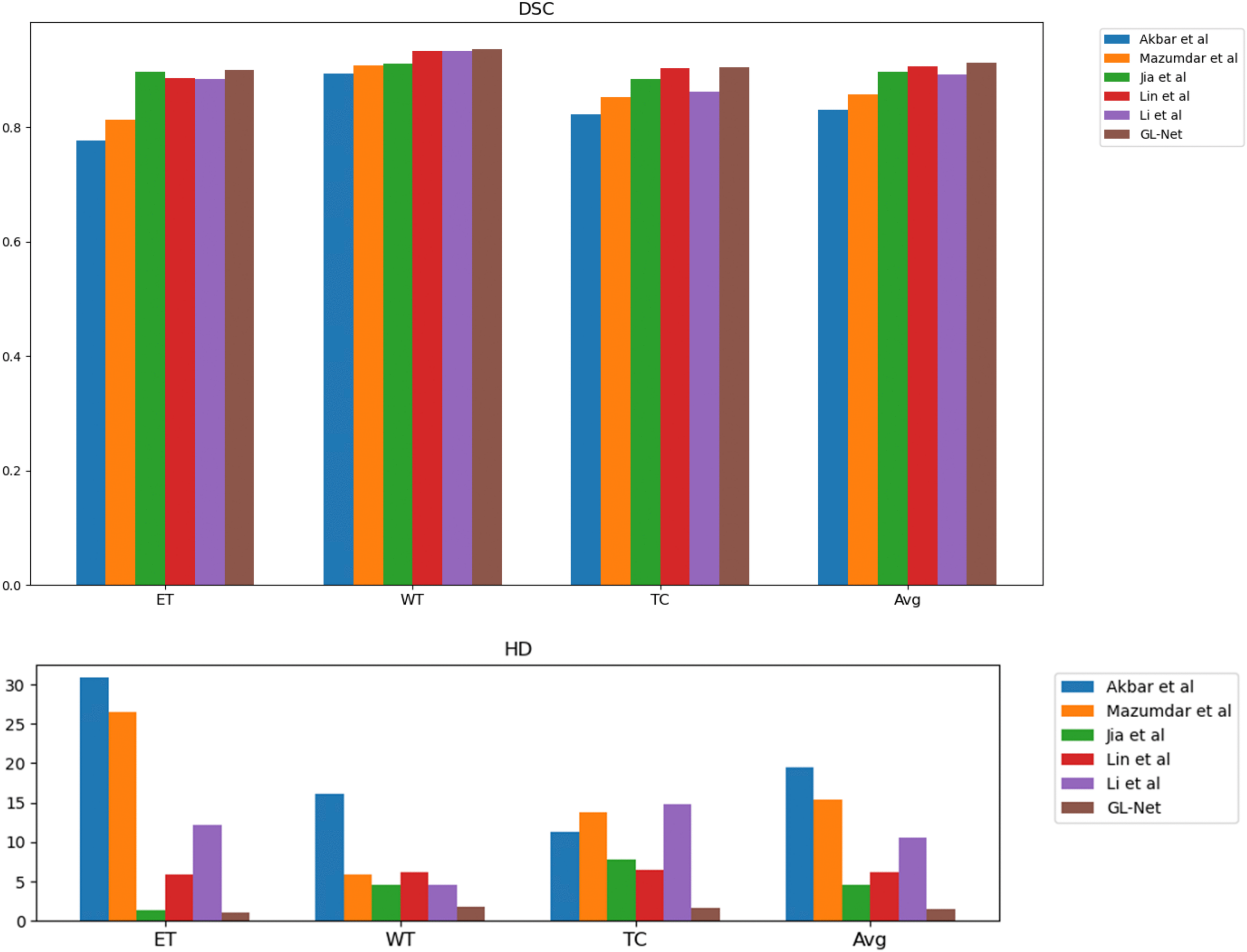
Bar chart of the comparison experiment results of GL-Net with other models on the BraTS2021.

First, the proposed GL-Net is evaluated on the internal hold-out test sets partitioned from the BraTS2019 and BraTS2021 official training data, and the results are compared with advanced algorithms, as shown in Tables 1 and 2 (Figs 8 and 9). It is important to note that the quantitative results of the comparative models presented in these tables are directly cited from their respective original publications rather than re-implemented in our exact experimental environment. While this provides a comprehensive contextual benchmark against current state-of-the-art methods, we explicitly acknowledge that these constitute indirect comparisons rather than head-to-head evaluations within a unified framework. Differences in preprocessing methods, training pipelines, and specific cross-validation splits can significantly impact performance metrics. Therefore, these comparative results should be interpreted with caution. Consequently, rather than claiming absolute superiority, we frame our results as highly competitive, noting that a definitive benchmarking would require re-implementing all models under an identical pipeline. In the BraTS2019, GL-Net achieves DSC scores of 0.855, 0.916, and 0.860 for the ET, WT, and TC regions, respectively. Notably, the DSC scores for the ET and WT regions are the highest. Additionally, GL-Net achieves HD values of 1.51 mm, 2.39 mm, and 1.59 mm for the ET, WT, and TC regions, respectively, with these HD values comparing favorably against current segmentation algorithms. Since the boundary between the TC and WT regions is relatively vague in some glioma datasets, GL-Net, compared to reference [23], lacks an edge detection module and thus does not extract edge features effectively. As a result, its performance in the TC region is inferior to that in reference [23], but GL-Net shows significant improvements in the ET and WT regions. Compared to the TransConver model [22], which is based on transformers and CNNs, GL-Net shows favorable results across most segmentation metrics, with particularly good performance in the ET region. In the BraTS2021 internal test set, GL-Net achieves DSC scores of 0.899, 0.936, and 0.905 for the ET, WT, and TC regions, respectively. Furthermore, GL-Net achieves HD values of 1.12 mm, 1.85 mm, and 1.68 mm for the ET, WT, and TC regions, respectively. Compared to the dual-branch network model based on attention mechanisms and super-resolution reconstruction (Jia et al.), GL-Net achieves highly comparable or superior metrics relative to the reported results of this algorithm, particularly in the segmentation of the WT and TC regions. Compared to the DenseTrans model proposed by Li et al. [18], GL-Net achieves excellent segmentation performance in the ET and TC regions, with further improvements in the WT region as well.

**Table 1:** The comparative experimental results of the GL-Net with other models on the BraTS2019.

Author	DSC				HD			
	ET	WT	TC	Avg	ET	WT	TC	Avg
Schlemper et al [12]	0.760	0.888	0.772	0.806	5.20	7.76	8.26	7.07
Akbar et al [13]	0.742	0.885	0.810	0.812	6.67	10.25	10.83	9.25
Liang et al [22]	0.789	0.859	0.838	0.828	2.69	2.59	1.61	2.29
Wang et al [21]	0.789	0.900	0.819	0.836	3.74	5.64	6.05	5.14
Rehman et al [36]	0.763	0.884	0.814	0.820	–	–	–	–
Zhu et al [23]	0.838	0.916	0.892	0.882	3.08	3.87	5.12	4.02
Chang et al [37]	0.782	0.890	0.812	0.828	3.82	8.53	7.43	6.59
AboElenein et al [38]	0.808	0.866	0.858	0.844	9.50	11.20	8.01	9.57
**GL-Net**	**0.855**	**0.916**	**0.860**	**0.877**	**1.51**	**2.39**	**1.59**	**1.83**

DSC — Dice Similarity Coefficient; HD — Hausdorff Distance. The performance metrics of the compared models are directly cited from their original publications.

**Table 2:** The comparative experimental results of the GL-Net with other models on the BraTS2021.

Author	DSC				HD			
	ET	WT	TC	Avg	ET	WT	TC	Avg
Akbar et al [13]	0.777	0.893	0.822	0.830	30.90	16.09	11.34	19.44
Mazumdar et al [39]	0.812	0.907	0.852	0.857	26.47	5.94	13.83	15.41
Jia et al [14]	0.896	0.911	0.883	0.896	1.41	4.58	7.81	4.60
Lin et al [16]	0.885	0.933	0.902	0.906	5.93	6.20	6.54	6.22
Li et al [18]	0.883	0.932	0.862	0.892	12.20	4.58	14.80	10.52
**GL-Net**	**0.899**	**0.936**	**0.905**	**0.913**	**1.12**	**1.85**	**1.68**	**1.55**

DSC — Dice Similarity Coefficient; HD — Hausdorff Distance. The performance metrics of the compared models are directly cited from their original publications.

**Fig 10:**
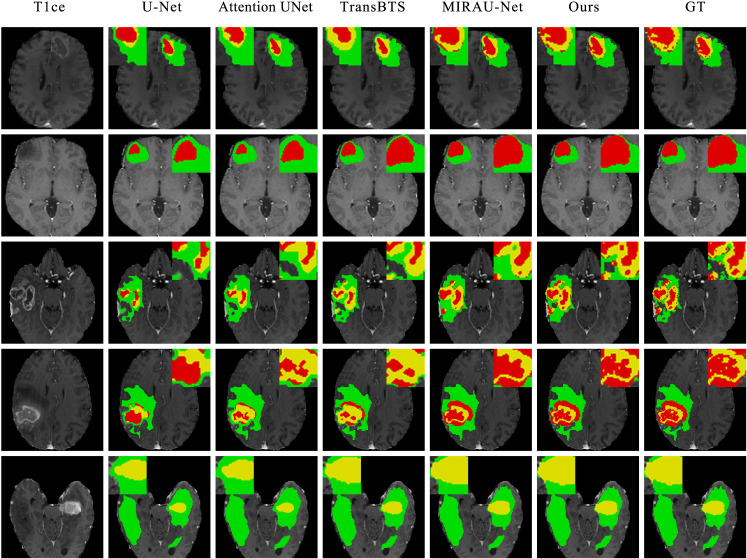
Examples of segmentation results on the BraTS2019 for the GL-Net, compared with other algorithms. WT: green + red + yellow, TC: red + yellow, ET: yellow.

In summary, GL-Net performs excellently in both DSC and HD metrics, demonstrating highly competitive segmentation optimization capabilities alongside current mainstream segmentation algorithms. This is due to GL-Net’s effective utilization of secondary features and further extraction through the LRM, which enhances the segmentation performance for brain gliomas. Fig 10 presents a visual comparison of the segmentation results for brain gliomas obtained by different algorithms. By comparing with the Ground Truth (GT), GL-Net achieves more accurate segmentation results than other algorithms, further validating the superior segmentation performance of the proposed algorithm from a qualitative analysis perspective.

### Ablation experiments

**Fig 11:**
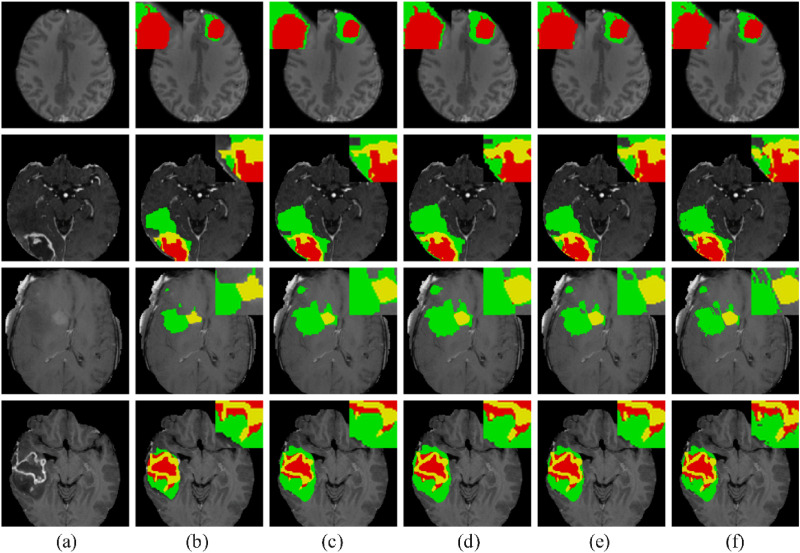
Examples of the ablation experiment results of this algorithm on the BraTS2019 dataset. **(a)**: T1CE, **(b)**: T – CNN, **(c)**: T – CNN + GGM, **(d)**: T – CNN + LRM, **(e)**: T – CNN + GGM + LRM, **(f)**: GT; WT: green + red + yellow, TC: red + yellow, ET: yellow.

Tables 3 and 4 display the quantitative ablation results of GL-Net on the BraTS2019 and BraTS2021 datasets, respectively, reported as Mean ± SD. To ensure absolute statistical rigor and account for potential non-normal distributions in the case-level metric differences, we report *p*-values from both the paired Student’s t-test and the non-parametric Wilcoxon signed-rank test (presented in parentheses). It can be observed that each of the proposed modules contributes to further improvements in segmentation performance. Crucially, both statistical tests reveal that the full GL-Net architecture (ALL) achieves highly significant improvements (*p* < 0.01) over the baseline T-CNN across almost all subregions in both DSC and HD metrics. While the individual addition of either the Gaussian Gating Module (GGM) or the Layered Refinement Module (LRM) noticeably enhances the segmentation of brain gliomas, the full model consistently yields statistically superior and more stable results (indicated by smaller standard deviations). This confirms that the performance gains are robust across the patient volumes and not driven by random variance. Fig 11 presents a comparison of the ablation study results for this algorithm. From the figure, it can be observed that, after incorporating the Gaussian Gating Module, inactive regions are effectively activated, enhancing the recognition of blurred edges. Furthermore, after adding the LRM, the segmentation performance of the WT, TC, and ET regions becomes more closely aligned with the ground truth labels.

**Table 3:** Ablation experiment results of GL-Net on the BraTS2019.

Model	Region	DSC	HD
T-CNN	ET	0.827±0.031(*p* < 0.01,*p* < 0.01)	3.51±0.55(*p* < 0.01,*p* < 0.01)
	WT	0.878±0.028(*p* < 0.01,*p* < 0.01)	4.88±0.72(*p* < 0.01,*p* < 0.01)
	TC	0.836±0.030(*p* < 0.01,*p* < 0.01)	3.79±0.61(*p* < 0.01,*p* < 0.01)
T-CNN + GGM	ET	0.845±0.027(*p* < 0.01,*p* < 0.01)	2.25±0.42(*p* < 0.01,*p* < 0.01)
	WT	0.893±0.025(*p* < 0.01,*p* < 0.01)	3.15±0.51(*p* < 0.01,*p* < 0.01)
	TC	0.856±0.028(0.10,0.08)	2.65±0.45(*p* < 0.01,*p* < 0.01)
T-CNN + LRM	ET	0.850±0.025(0.04,0.06)	2.01±0.38(*p* < 0.01,*p* < 0.01)
	WT	0.897±0.024(*p* < 0.01,*p* < 0.01)	2.95±0.48(*p* < 0.01,*p* < 0.01)
	TC	0.848±0.026(*p* < 0.01,*p* < 0.01)	2.81±0.49(*p* < 0.01,*p* < 0.01)
ALL	ET	**0.855±0.022**	**1.51±0.30**
	WT	**0.916±0.020**	**2.39±0.35**
	TC	**0.860±0.024**	**1.59±0.32**

5-fold cross-validation results are presented as Mean ± Standard Deviation (SD). Values in parentheses $\left(p_{\text{t-test}},p_{\text{wilcoxon}}\right)$ indicate *p*-values from the paired Student’s t-test and the non-parametric Wilcoxon signed-rank test, respectively, comparing the respective baseline model against the final proposed model (ALL / DiceBRD loss). A value of (*p* < 0.01) denotes *p* < 0.01. Extremely small *p*-values are reported as *p* < 0.01 following standard statistical reporting guidelines.

**Table 4:** Ablation experiment results of GL-Net on the BraTS2021.

Model	Region	DSC	HD
T-CNN	ET	0.855±0.029(*p* < 0.01,*p* < 0.01)	3.58±0.58(*p* < 0.01,*p* < 0.01)
	WT	0.883±0.027(*p* < 0.01,*p* < 0.01)	4.88±0.65(*p* < 0.01,*p* < 0.01)
	TC	0.869±0.028(*p* < 0.01,*p* < 0.01)	4.03±0.62(*p* < 0.01,*p* < 0.01)
T-CNN + GGM	ET	0.870±0.025(*p* < 0.01,*p* < 0.01)	2.45±0.45(*p* < 0.01,*p* < 0.01)
	WT	0.910±0.023(*p* < 0.01,*p* < 0.01)	3.92±0.54(*p* < 0.01,*p* < 0.01)
	TC	0.891±0.024(*p* < 0.01,*p* < 0.01)	3.64±0.51(*p* < 0.01,*p* < 0.01)
T-CNN + LRM	ET	0.881±0.023(*p* < 0.01,*p* < 0.01)	2.13±0.39(*p* < 0.01,*p* < 0.01)
	WT	0.918±0.022(*p* < 0.01,*p* < 0.01)	2.87±0.42(*p* < 0.01,*p* < 0.01)
	TC	0.884±0.025(*p* < 0.01,*p* < 0.01)	2.56±0.40(*p* < 0.01,*p* < 0.01)
ALL	ET	**0.899±0.020**	**1.12±0.25**
	WT	**0.936±0.018**	**1.85±0.31**
	TC	**0.905±0.021**	**1.68±0.28**

5-fold cross-validation results are presented as Mean ± Standard Deviation (SD). Values in parentheses $\left(p_{\text{t-test}},p_{\text{wilcoxon}}\right)$ indicate *p*-values from the paired Student’s t-test and the non-parametric Wilcoxon signed-rank test, respectively, comparing the respective baseline model against the final proposed model (ALL / DiceBRD loss). Extremely small *p*-values are reported as *p* < 0.01 following standard statistical reporting guidelines.

**Fig 12:**
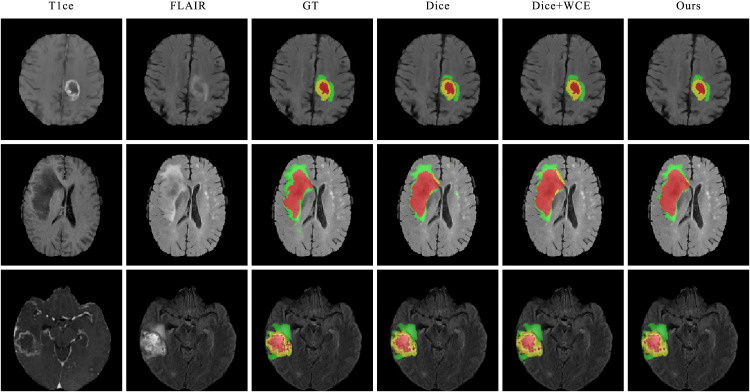
Examples of segmentation results of the loss function proposed on the BraTS2019. WT: green + red + yellow, TC: red + yellow, ET: yellow.

To verify the effectiveness of the proposed loss function, ablation experiments were conducted. Table 5 presents the results obtained from training GL-Net with various loss functions. First, a comparison was made between Dice loss and Dice+WCE, where Dice+WCE improved the DSC by 0.5%, 0.8%, and 0.4% for the ET, WT, and TC regions, respectively, and reduced HD by 0.6 mm, 0.8 mm, and 0.84 mm, respectively. This indicates that WCE effectively mitigates the class imbalance problem. WCE refers to the use of a distance map for weighting and includes all voxels in the loss calculation. In the comparison between Dice+WCE and the proposed DiceBRD loss function, the proposed loss improved the DSC by 0.6%, 1.2%, and 0.4% for the ET, WT, and TC regions, respectively, while reducing HD by 0.18 mm, 0.61 mm, and 0.42 mm, respectively. More importantly, the dual statistical analyses rigorously validate the specific advantages of the DiceBRD loss. While the DSC improvements over Dice+WCE show moderate statistical significance (e.g., *p* = 0.03 for both t-test and Wilcoxon test in WT), the reductions in the Hausdorff Distance (HD) are highly significant across all subregions (p≤0.01 for ET, *p* < 0.01 for WT and TC in both tests). This explicitly confirms our methodological hypothesis: by strictly restricting the weighted cross-entropy calculation to the dynamically evolving boundary regions, the model successfully tightens the geometric contour alignment, leading to a statistically robust and reliable reduction in boundary errors (HD). Additionally, qualitative results, as shown in Fig 12, indicate that the proposed loss function enhances network performance and yields satisfactory experimental results, which is consistent with the quantitative comparisons in Table 5.

**Table 5:** Comparative experimental results of different loss functions for GL-Net on the BraTS2021 internal test set (via 5-fold cross-validation).

Method	Region	DSC	HD
Dice	ET	0.846±0.034(0.02,0.02)	2.23±0.55(*p* < 0.01,*p* < 0.01)
	WT	0.899±0.031(*p* < 0.01,*p* < 0.01)	3.42±0.71(*p* < 0.01,*p* < 0.01)
	TC	0.856±0.022(0.02,0.01)	2.78±0.62(*p* < 0.01,*p* < 0.01)
Dice+WCE	ET	0.851±0.029(0.16,0.21)	1.63±0.42(0.01,0.01)
	WT	0.907±0.028(0.03,0.03)	2.62±0.52(*p* < 0.01,*p* < 0.01)
	TC	0.860±0.027(0.32,0.24)	1.94±0.48(*p* < 0.01,*p* < 0.01)
DiceBRD loss	ET	**0.857±0.024**	**1.45±0.32**
	WT	**0.919±0.022**	**2.01±0.38**
	TC	**0.864±0.025**	**1.52±0.35**

5-fold cross-validation results are presented as Mean ± Standard Deviation (SD). Values in parentheses $\left(p_{\text{t-test}},p_{\text{wilcoxon}}\right)$ indicate *p*-values from the paired Student’s t-test and the non-parametric Wilcoxon signed-rank test, respectively, comparing the respective baseline model against the final proposed model (ALL / DiceBRD loss). Extremely small *p*-values are reported as *p* < 0.01 following standard statistical reporting guidelines.

It is worth noting that these findings may still be influenced by characteristics of the training data. The BraTS datasets, although standardized and widely adopted, remain limited in size compared with real-world multi-center cohorts, which may constrain the generalizability of the observed improvements. Moreover, the significant class imbalance inherent to glioma subregions—particularly the small volume of the ET region—may amplify sensitivity to sampling variations, potentially affecting the magnitude of performance gains. In addition, the curated nature of BraTS introduces a degree of selection bias, meaning the effectiveness of the proposed loss function on more heterogeneous clinical data remains to be further validated. These factors should be considered when interpreting the ablation results.

### VASARI-based feature analysis

While geometric metrics such as DSC and HD provide quantitative measures of spatial overlap and boundary distances, they do not fully capture the clinical utility of a segmentation model. In clinical workflows, the ultimate goal of tumor delineation is to extract reliable morphological and compositional metrics—such as the proportions of necrosis, enhancing, and non-enhancing tumor regions—that directly inform diagnosis and treatment planning. Therefore, downstream classification consistency serves as a crucial indicator of potential clinical relevance for segmentation quality. If the segmentation masks generated by GL-Net yield VASARI features and subsequent GBM diagnostic performance that are statistically indistinguishable from those derived from expert manual annotations (ground truth), it provides valuable complementary and indirect evidence that GL-Net successfully preserves the clinically critical morphological variations and subregion proportions required for accurate diagnosis, thereby bridging the gap between pixel-level accuracy and potential clinical relevance.

**Fig 13:**
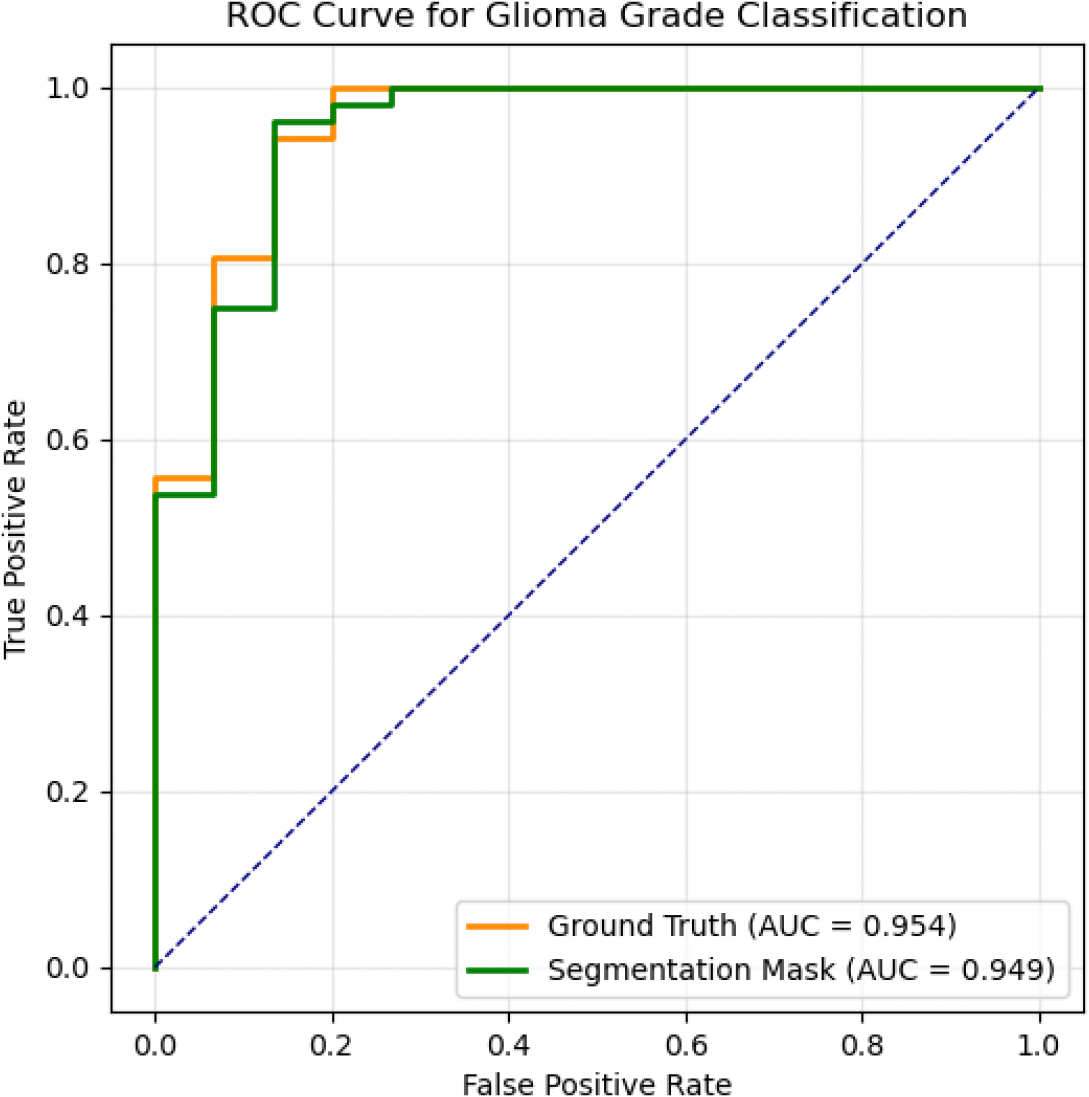
ROC curves for GBM classification using GL-Net and BraTS segmentation masks.

Following this rationale, after extracting VASARI structural imaging features from both the GL-Net-generated masks and the BraTS gold-standard masks, we constructed GBM classification models and compared the discriminative performance of the two feature sets. The results (Table 6) showed that the model based on the gold-standard masks achieved an AUC of 0.954, ACC of 0.940, and F1-score of 0.962, while the model based on GL-Net outputs achieved an AUC of 0.949, ACC of 0.925, and F1-score of 0.952, indicating highly consistent performance across all metrics (Fig 13). DeLong test further confirmed that the difference in AUC between the two models was not statistically significant (p = 0.9996).

**Table 6:** GBM classification performance using GL-Net vs. BraTS masks.

Mask	AUC	ACC	F1	SEN	SPE	PRE
BraTS seg	0.954	0.940	0.962	0.981	0.800	0.944
GL-Net	0.949	0.925	0.952	0.962	0.800	0.943

SEN — Sensitivity; SPE — Specificity; PRE — Precision; BraTS seg — BraTS gold-standard segmentation masks.

**Fig 14:**
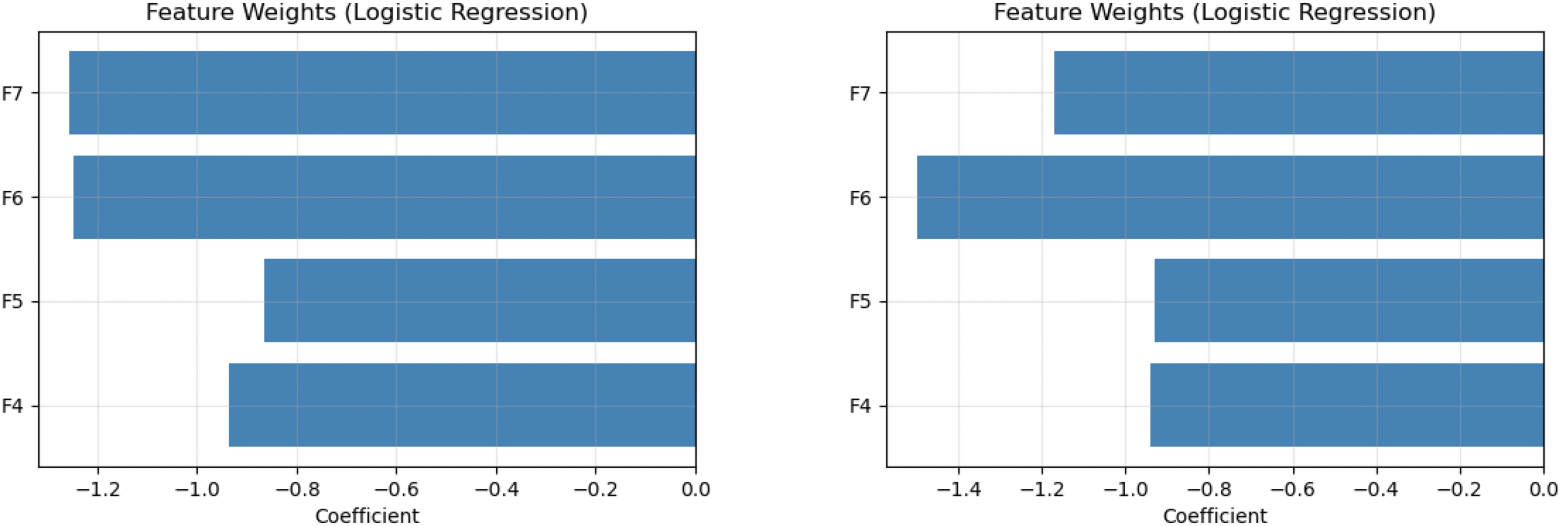
Feature weight comparison between GL-Net (left) and gold-standard masks (right).

In addition, visualization of the logistic regression feature weights (Fig 14) revealed that the weight distribution patterns of both models were almost identical, further demonstrating that the structural imaging features extracted from GL-Net segmentations contribute to GBM classification comparably to manual annotations. Collectively, these results suggest that GL-Net segmentation masks can serve as a stable and reliable input for GBM imaging feature extraction and diagnostic classification, offering indirect evidence of the model’s potential clinical relevance rather than definitive proof of absolute segmentation accuracy.

## Conclusion

Medical imaging plays a pivotal role in clinical diagnosis, particularly for the segmentation of brain glioma in MRI. Despite the promise of deep learning, performance is often limited by data scarcity and image complexity. In this study, we proposed GL-Net, a brain glioma segmentation algorithm that integrates deep learning with prior knowledge. The Gaussian Gating Module (GGM) effectively suppresses image noise, activates secondary features, and enhances recognition of blurred edges, while the Label Refinement Module (LRM) leverages label priors for multi-scale feature fusion, improving the perception of fine details. A dynamic weighted loss function was also designed to emphasize edge-region voxels, mitigating the class imbalance problem.

Extensive experiments on the BraTS2019 and BraTS2021 datasets demonstrate that GL-Net achieves robust and highly competitive performance, with average DSCs of 0.877 and 0.913, and HDs of 1.83 mm and 1.55 mm, respectively, achieving highly competitive performance relative to reported benchmarks, particularly in the segmentation of the Enhancing Tumor (ET) and Whole Tumor (WT) regions. To assess clinical relevance, VASARI-based structural imaging features were extracted from GL-Net segmentations and compared to features derived from BraTS gold-standard masks. GBM classification results showed no statistically significant difference (p = 0.9996) between the two feature sets, and logistic regression feature weight distributions were nearly identical, suggesting that GL-Net provides stable and clinically meaningful representations comparable to manual annotations, thus offering complementary evidence of its potential clinical relevance.

While GL-Net demonstrates strong segmentation accuracy and reliable downstream feature extraction, claims regarding its direct clinical applicability must be interpreted with caution. A primary limitation of the current study is the reliance on the highly curated BraTS datasets. Although multi-institutional, these standardized datasets may not fully capture the extreme heterogeneity, unpredictable artifacts, and diverse scanning protocols encountered in independent, real-world clinical cohorts. The absence of external validation on an independent real-world dataset means the model’s operational robustness in everyday clinical practice remains to be definitively proven. Furthermore, to address the limitations of citing unstandardized comparative results, future work will involve re-implementing state-of-the-art baseline models within a unified training, preprocessing, and evaluation framework (such as nnU-Net) to enable standardized, head-to-head benchmarking. Therefore, extensive external validation on independent clinical cohorts and subsequent prospective trials are essential next steps to rigorously evaluate the model’s true generalizability. Future work will focus on conducting these prospective validations, optimizing GL-Net for small or poorly defined tumors, and iteratively advancing its robust incorporation into clinical workflows to truly enhance diagnostic efficiency and reliability.
