# Supplementary material for: GL-Net: A knowledge-guided Gaussian-gated and layered refinement network for 3D MRI segmentation of brain gliomas
Source: PLoS One. 2026 Jun 22;21(6):e0351953. doi: 10.1371/journal.pone.0351953 (PMC13286167; doi:10.1371/journal.pone.0351953)
Supplement: Attachment — Submitted filename: Report_PONE-D-25-43663_R1_17.02.26 .pdf [file pone.0351953.s003.pdf]

Axioms PONE-D-25-43663_R1
Report

The manuscript proposes GL-Net, a deep learning framework for brain glioma segmentation from MRI that incorporates prior knowledge through a Gaussian Gating Module (GGM) and a Label Refinement Module (LRM), together with a dynamic weighted loss function to address class imbalance. The methodological design is clearly motivated, and the individual components are well integrated within the network architecture.

Experimental results on the BraTS2019 and BraTS2021 datasets demonstrate competitive and in several cases superior performance compared to existing methods, particularly for Enhancing Tumor and Whole Tumor regions. The additional evaluation of clinical relevance, based on VASARI feature extraction and GBM classification, is a strong aspect of the work and supports the claim that the proposed segmentations are comparable to expert annotations.

The study is technically sound and well executed. Nevertheless, further validation on larger, multi-center datasets would be important to assess robustness and generalizability. Additional clarification regarding performance on small or poorly defined tumors, as well as further discussion of potential integration into routine clinical workflows and expert assessment pipelines, as noted by the authors in the Conclusion, would be beneficial.

Overall, the manuscript addresses a relevant problem in medical image analysis and meets the technical standards expected for publication. In my view, the authors have responded satisfactorily to the referees' comments. However, an important aspect remains insufficiently addressed in both the original and revised versions of the manuscript, namely the **role of environmental noise** in the proposed approach and a comparison with results obtained from **mathematical and biomechanical modeling frameworks**.

In this context, a more comprehensive and in-depth discussion of the following points would be beneficial before the manuscript can be considered for publication:

1. Comparison with existing approaches

The manuscript would benefit from a clearer positioning of the proposed approach with respect to existing methods, particularly in relation to the following published works, which the authors are encouraged to discuss and compare in detail:

i) "*Glioblastoma survival prediction through MRI and clinical data integration with transfer learning*", Int. J. CARS (2025), **https://doi.org/10.1007/s11548-025-03548-1**, which employs a deep learning architecture for MRI-based glioma segmentation and feature extraction using latent-space representations.

ii) "*Biomechanical modeling of glioblastoma progression: a comprehensive review from classic mathematical frameworks to data-driven strategies*", Biomechanics and Modeling in Mechanobiology (2026) **25**:1, **https://doi.org/10.1007/s10237-025-02028-4**, which reviews the evolution of glioblastoma modeling from reaction–diffusion equations to continuum biomechanics, multiphysics approaches, and hybrid data-driven models.

iii) "*Quantifying the Growth of Glioblastoma Tumors Using Multimodal MRI Brain Images*", Cancers (Basel). 2023, **15**:3614. **doi: 10.3390/cancers15143614**, where tumor segmentation and radiomics are combined to predict glioblastoma growth and survival.

iv) "*Heterogeneous biomechanical/mathematical modeling of spatial prediction of glioblastoma progression using MRI-based finite element methods*", Computer Methods and Programs in Biomedicine, **257**, 108441 (2024), which integrates reaction–diffusion modeling with MR imaging.

v) "*Stochastic modeling of glioblastoma spread: a numerical simulation study*", 13th International Workshop on Innovative Simulation for Healthcare, 011, 21st International Multidisciplinary Modeling & Simulation Multiconference 2724-0371 (2024); doi: 10.46354/i3m.2024.iwish.011, where the authors use a mesoscopic modeling by considering the stochastic fluctuations of tumor behavior.

2. Related literature on stochastic and population-based models

The discussion would also benefit from citing and commenting on relevant literature concerning cancer growth dynamics, stochastic reaction–diffusion models, and population dynamics, including representative works in stochastic biological and ecological modeling: i) "Stochastic models for phytoplankton dynamics in Mediterranean Sea", Ecological Complexity **27**, 84-103 (2016); ii) "*Coexistence of Resonant Activation and Noise Enhanced Stability in a Model of Tumor-Host Interaction: Statistics of Extinction Times*", Acta Physica Polonica B, **37**, 1651-1666 (2006); iii) "*Stochastic dynamics of leukemic cells under an intermittent targeted therapy*", Theory in Biosciences **130**, 203-210 (2011); iv) "*Ecological complex systems*", Eur. Phys. J. B **65**, 307-314 (2008).

3. Role of environmental noise and fluctuations

Environmental noise and random fluctuations are fundamental components of complex systems such as glioblastoma. Given their recognized importance in physical, biological, and mathematical models of cancer growth, it would be useful to include a short paragraph in the Introduction discussing the constructive role of noise and fluctuations in complex systems. This discussion could be framed more broadly, for example in connection with the importance of fluctuations emphasized in Giorgio Parisi's Nobel Prize in Physics (2021), and supported by appropriate references from the physics and biological modeling literature: i) Parisi G., Nobel Lecture: Multiple Equilibria, Rev. Mod. Phys. **95**, 030501 (2023), which highlights the impact of fluctuations across physics; ii) Phys. Rev. A **92**, 013830 (2015), which explores the constructive role of noise in quantum systems; iii) Eur. Phys. J. B **70**, 145-151 (2009), which highlight the positive role of noise in classical systems, and iv) Eur. Phys. J. B **65**, 453-458 (2008), which shows the positive role of noise in biological systems.

Including and discussing these contributions would help place the manuscript in a broader and more physically meaningful context.

In summary, a more explicit consideration of **environmental noise and stochastic effects**—potentially through comparison with or incorporation of stochastic reaction–diffusion or biomechanical models—would help place the proposed method in a broader and more physically meaningful context and strengthen the overall manuscript.
