# Supplementary material for: GL-Net: A knowledge-guided Gaussian-gated and layered refinement network for 3D MRI segmentation of brain gliomas
Source: PLoS One. 2026 Jun 22;21(6):e0351953. doi: 10.1371/journal.pone.0351953 (PMC13286167; doi:10.1371/journal.pone.0351953)
Supplement: Attachment — Submitted filename: Response to Reviewers.docx [file pone.0351953.s001.docx]

**Response to Reviewers**

Dear Editors and Reviewers,

We express our sincere gratitude to you and your colleagues for reviewing our submitted manuscript and providing valuable comments and suggestions. We highly appreciate these invaluable suggestions and believe they have played a crucial role in our research. We have carefully considered each of your suggestions, conducting thorough revisions to ensure the academic and methodological accuracy and credibility of our paper. Here, we sincerely respond to your review comments, hoping that our responses meet your expectations. Once again, we thank you for your valuable feedback and patience. Below are our responses to the specific comments:

**Additional Editor Comments:**

“This manuscript presents a technically interesting and potentially impactful approach to 3D glioblastoma segmentation using a hybrid transformer–CNN architecture. However, its clinical positioning remains underdeveloped. The authors are strongly encouraged to better contextualize their model within current clinical practice by integrating relevant literature and aligning their discussion with existing radiological evaluation frameworks. Further comparison with prior models will also be necessary to objectively assess the proposed method’s contribution to the field.

Title:

The title is clear and informative, but could be further strengthened by explicitly highlighting the methodological innovation introduced in the paper, to better convey its novelty and relevance compared to existing literature.

Abstract:

The abstract is well written and provides a coherent summary of the study. However, it would benefit from the inclusion of more specific performance results and a brief mention of the potential clinical implications of the proposed method, in order to emphasize its real-world utility.

Introduction:

The introduction is well structured and successfully establishes the clinical relevance of accurate glioblastoma segmentation. That said, to improve scientific depth and contextual clarity, the background section should be expanded to include more details on current radiological assessment frameworks commonly used in clinical practice for glioma characterization. Including references to standardized descriptors would strengthen the clinical foundation of the work and better justify the need for automated approaches.

Materials and Methods:

The methodology is described in sufficient detail. However, clarification is needed on whether the dataset reflects adequate clinical and technical heterogeneity (e.g., use of different MRI scanners and acquisition protocols), to ensure that the proposed model generalizes well across varying real-world conditions.

Statistical Analysis:

The statistical analysis is sound and appropriately rigorous. Nonetheless, the authors should discuss potential limitations related to dataset size, class imbalance, or selection bias, and how these factors may have influenced the results.

Results:

The results are clearly presented. However, a more direct and quantitative comparison with existing models is encouraged—ideally through a summary table—to help readers appreciate the degree of improvement introduced by the proposed method.

Discussion:

The discussion effectively highlights the main contributions of the work, but it would benefit from a more critical integration of recent literature, particularly studies that bridge conventional MRI evaluation and computational methods. Including references to structured radiological reporting tools and lexicons currently used in glioma assessment would enhance the discussion and help demonstrate how the proposed model might complement existing clinical workflows.

Conclusion:

The conclusions are consistent with the reported results. However, they should more explicitly acknowledge that implementation in clinical practice requires further validation, particularly in relation to how the model correlates with standard radiological evaluation criteria. The authors should stress that clinical translation depends on how well the algorithm integrates with expert visual assessment and standardized MRI descriptors.”

**Reply to Additional Editor:**

It is our great honor that our work has been recognized by the editors, and we sincerely appreciate the constructive comments provided. Below, we provide our responses to the suggestions for each section.

**Response regarding the title:**

To better highlight the methodological innovation and emphasize the novelty of our approach, we have revised the title to explicitly reflect the integration of prior knowledge and the key technical modules (Gaussian-Gated and Layered Refinement). The revised title now reads: “GL-Net: A Knowledge-Guided Gaussian-Gated and Layered Refinement Network for 3D MRI Segmentation of Brain Gliomas.” This modification better conveys the methodological contribution of our work while keeping the title concise and informative.

**Response regarding the abstract:**

To better emphasize the real-world applicability and performance of our proposed method, we have revised the abstract to include the quantitative analysis results based on VASARI features extracted from the segmentation outputs. We also briefly highlighted the potential clinical significance of GL-Net in supporting accurate glioma diagnosis and treatment planning. The revised abstract now provides clearer evidence of the model’s effectiveness and clinical relevance.

**Response regarding Materials and Methods:**

In the revised section, we have added a detailed description of the datasets used, including data sources, imaging device manufacturers, magnetic field strength ranges, and relevant imaging protocols. These additions ensure that the proposed model can be well generalized across diverse real-world conditions.

**Response regarding statistical analysis:**

This is an excellent suggestion. The issues raised—such as dataset size, class imbalance, and selection bias—are indeed among the key challenges that our work aims to address. Following the editors’ recommendation, we have incorporated additional content in the Results section to specifically discuss these aspects.

**Response regarding the Results:**

We have optimized the presentation of the summary tables and added new figures to illustrate the results. We believe these additions will help readers more intuitively understand the extent of improvements achieved by the proposed method.

**Response regarding the Discussion and Conclusion:**

In these two sections, we have added content on the quantitative VASARI evaluation of segmentation masks produced by our model compared with the gold-standard masks delineated by clinicians. The results demonstrate that the proposed model holds substantial potential and utility in supporting clinical practice and subsequent research.

**Response regarding Figures, Tables, and Diagrams:**

In the Methods section, we have added a workflow diagram to illustrate how the model can be integrated into the clinical workflow.

**Response regarding References:**

We have added new references in the manuscript concerning the use of structured MRI descriptors and the VASARI visual assessment system for glioma grading.

**Reviewer #1:** I suggest including in the discussion or in the conclusion that the proposed method for the automatic 3D segmentation of brain gliomas must be implemented in daily clinical practice and must be correlated with the clinical radiological assessment based on the identification of various MRI characteristics, first and foremost those of the conventional MRI examination, such as those codified by the so-called VASARI lexicon.

**Reply to Reviewer 1:**

Thank you very much for your careful review and approval of our paper. The suggestions and viewpoints you provided have played a crucial guiding role in our research. Here are our responses to specific comments:

Following your suggestion, we conducted additional experiments to further evaluate the clinical applicability of our method. Specifically, we extracted F4–F7 structural imaging features based on the VASARI scoring system from both GL-Net segmentation outputs and the gold-standard masks. This allowed us to assess the consistency and reliability of the model’s segmentation results in the context of GBM clinical characterization, and to verify that the model’s support for clinical diagnosis is comparable to manual annotation.

The results showed that, after extracting VASARI structural imaging features from both GL-Net and BraTS gold-standard masks, we built GBM classification models and compared the discriminative performance of the two feature sets. The model based on gold-standard masks achieved an AUC of 0.954, ACC of 0.940, and F1 score of 0.962, whereas the model based on GL-Net outputs achieved an AUC of 0.949, ACC of 0.925, and F1 score of 0.952, demonstrating a high degree of consistency across all metrics. DeLong’s test further indicated that the difference in AUC between the two models was not statistically significant (p = 0.9996).

Additionally, we visualized the feature weights of the logistic regression models (Figure X) and observed that the weight distribution patterns were nearly identical between the two models. This further confirms that the structural imaging features derived from GL-Net segmentations contribute to GBM classification in a manner highly consistent with manual annotations.

In summary, the GL-Net segmentation masks provide stable and reliable structural imaging features for GBM clinical feature extraction and diagnostic discrimination, demonstrating comparable performance to manual gold-standard masks. These findings indicate that GL-Net has strong potential for integration into daily clinical practice, supporting radiological assessment based on MRI characteristics, including those codified by the VASARI lexicon, and facilitating accurate and efficient clinical decision-making.

Sincerely,

Huimin Lu
