# Supplementary material for: GL-Net: A knowledge-guided Gaussian-gated and layered refinement network for 3D MRI segmentation of brain gliomas
Source: PLoS One. 2026 Jun 22;21(6):e0351953. doi: 10.1371/journal.pone.0351953 (PMC13286167; doi:10.1371/journal.pone.0351953)
Supplement: Attachment — Submitted filename: Reveiwers suggestions for this essay.doc [file pone.0351953.s002.doc]

### Overall Comments

### This study presents the GL-Net framework, which integrates prior knowledge with a hybrid network structure to achieve excellent performance in glioma MRI segmentation. The clinical adaptability verification lays a solid foundation for practical applications, with a clear research logic and sufficient data support. However, refinements are needed in detail explanations and limitation discussions to further enhance the paper’s rigor and readability.

### Specific Revision Suggestions

1.Clarify the impact of equipment/magnetic field strength on performance: The manuscript mentions the dataset includes MRI devices from different manufacturers and varying magnetic field strengths, but does not specify whether these factors affect model performance. Briefly analyze the potential correlation between such heterogeneity and segmentation metrics (e.g., DSC, HD) in the Results or Discussion section, based on existing data without additional experiments.

2.Elaborate on the detailed reasons for inferior TC region performance: The manuscript notes that GL-Net’s performance in the TC region is slightly lower than some models, presumably due to the lack of an edge detection module. Further refine the reasons in the Discussion (e.g., the uniqueness of boundary features between TC and WT regions) and briefly propose optimization directions that do not require major model modifications (e.g., adjusting edge weight allocation), without supplementary comparative experiments.

3.Illustrate the specific impact of dataset limitations: The Discussion mentions selection bias and limited sample size in the BraTS dataset. Supplement qualitative explanations on how such limitations may affect the model’s generalization in real clinical scenarios (e.g., adaptability to rare tumor subtypes), without quantitative analysis.

4.Note the limitation of VASARI feature verification: Currently, clinical relevance is only verified based on F4-F7 features. Mention this limitation in the Discussion and illustrate the potential value of extending to the full VASARI feature set, without actually supplementing feature extraction experiments, just providing a prospective explanation.
