# Supplementary material for: GL-Net: A knowledge-guided Gaussian-gated and layered refinement network for 3D MRI segmentation of brain gliomas
Source: PLoS One. 2026 Jun 22;21(6):e0351953. doi: 10.1371/journal.pone.0351953 (PMC13286167; doi:10.1371/journal.pone.0351953)
Supplement: Attachment — Submitted filename: Response_to_Reviewers_auresp_2.docx [file pone.0351953.s004.docx]

**Response to Reviewers**

Dear Editors and Reviewers,

We express our sincere gratitude to you and your colleagues for reviewing our submitted manuscript and providing valuable comments and suggestions. We highly appreciate these invaluable suggestions and believe they have played a crucial role in our research. We have carefully considered each of your suggestions, conducting thorough revisions to ensure the academic and methodological accuracy and credibility of our paper. Here, we sincerely respond to your review comments, hoping that our responses meet your expectations. Once again, we thank you for your valuable feedback and patience. Below are our responses to the specific comments:

**1. Reviewer's Comment:** “While the manuscript reports improvements in DSC and HD metrics, no statistical significance testing is provided to support these performance gains. Given that some improvements are relatively modest in magnitude, it would be important to demonstrate whether the reported differences are statistically significant across folds. Reporting mean ± standard deviation and including appropriate paired statistical tests (e.g., paired t-test or Wilcoxon signed-rank test) would strengthen the robustness of the conclusions.”

**Response:** We sincerely thank the reviewer for this rigorous and crucial suggestion. We completely agree that relying solely on average metrics is insufficient to prove the absolute superiority of the model, and that statistical significance testing is essential to confirm that the observed improvements are robust across different cross-validation folds. Following your valuable advice, we have comprehensively updated our quantitative evaluation:

1. We have revised the result tables (specifically the ablation study tables, e.g., Tables 3, 4, and 5) to report all metrics in the format of **Mean ± Standard Deviation (SD)** across the five-fold cross-validation. Furthermore, we conducted **paired t-tests** to compare the baseline models with our proposed GL-Net. The resulting $p$-values are now included in the tables (values in parentheses), where $p<0.01$ indicates high statistical significance. The results confirm that our performance gains, even those with smaller margins, are indeed statistically significant.

2. To formally document this statistical evaluation, we have added a dedicated paragraph to the **“Dataset and evaluation metrics”** section in the revised manuscript: *“Furthermore, to rigorously validate the performance improvements of the proposed GL-Net, statistical significance testing was conducted. The quantitative segmentation results across the five-fold cross-validation are reported as Mean* $\pm$ *Standard Deviation (SD). A paired Student’s t-test was utilized to determine whether the performance differences between the baseline configurations (or comparative models) and the proposed GL-Net were statistically significant across the test folds. A* $p$*-value of* $<0.05$ *was considered statistically significant, and* $p<0.01$ *was considered highly significant.”*

3. In addition to updating the tables with Mean $\pm$ SD and $p$-values, we have also expanded the text in the **‘Ablation experiments’** section to deeply analyze these statistical findings. Most notably, in the analysis of our proposed DiceBRD loss function (Table 5), we explicitly highlighted that while the overall volume overlap (DSC) showed moderate improvements, the reductions in boundary errors (Hausdorff Distance) were highly statistically significant ($p\leq0.01$). This statistical evidence perfectly corroborates our methodological claim that the dynamic boundary weighting specifically and robustly refines fuzzy edge contours.

**2. Reviewer's Comment:** “Tables 1 and 2 compare GL-Net with several existing models. However, it is not clearly stated whether these models were re-implemented under identical preprocessing and cross-validation settings, or whether the results were directly cited from the original publications. This distinction is important for assessing fairness and reproducibility. Explicit clarification of the comparison protocol would improve methodological transparency.”

**Response:** We sincerely thank the reviewer for pointing out this important detail regarding methodological transparency. We completely agree that clarifying the evaluation protocol is crucial for fairness and reproducibility.

We would like to clarify that the quantitative results of the comparative models presented in Tables 1 and 2 were directly cited from their respective original publications, rather than being re-implemented under our exact preprocessing and cross-validation conditions. To ensure transparency, we have added explicit statements to the revised manuscript acknowledging this.

Specifically, we have updated the text in the "Comparative Experiments of GL-Net" section to read: *"It is important to note that the quantitative results of the comparative models presented in these tables are directly cited from their respective original publications rather than re-implemented in our exact experimental environment. While this provides a comprehensive benchmark against current state-of-the-art methods, we acknowledge that minor variations in data splits (e.g., cross-validation folds) and preprocessing pipelines among these original studies may exist."*

Additionally, we have added a clarifying note to the footer of both Table 1 and Table 2: *"The performance metrics of the* compared *models are directly cited from their original publications."*

**3. Reviewer's Comment:** “The mathematical formulation of the DiceBRD loss function requires clearer explanation. In particular, the relationship between the ROI-based region restriction and the combined Dice and weighted cross-entropy terms could be more explicitly described. Additionally, certain variable definitions appear repetitive or ambiguous. A more precise and structured presentation of the loss formulation would enhance technical clarity.”

**Response:** We sincerely thank the reviewer for this highly constructive and precise feedback. We agree that the mathematical formulation of the DiceBRD loss function in the original manuscript lacked sufficient rigor and contained typographical ambiguities.

In the revised manuscript, we have comprehensively restructured the presentation of the DiceBRD loss to ensure technical clarity:

1. We have corrected the typographical error where y_ic was ambiguously defined twice. It is now correctly defined as the ground truth one-hot encoding (y_ic∈{0,1}), while p_ic∈[0,1] is strictly defined as the predicted probability.

2. We replaced the vague terms N and voxels with standard set notations. We now use R to denote the dynamically extracted boundary region of interest, ∣R∣ for its cardinality (number of voxels), and V to denote the set of all voxels in the entire image volume.

3. We explicitly clarified the relationship between the two loss terms. The revised text now clearly states that the Dice loss term operates on the global domain V to capture overall topological structure and mitigate class imbalance, whereas the dynamic weighted cross-entropy term (L_BRD) is strictly restricted to the local boundary domain R (i∈R). This decoupling explains why the combination is highly effective at refining fuzzy edges without losing global context.

**4. Reviewer's Comment:** “The manuscript evaluates segmentation performance indirectly through downstream GBM classification using selected VASARI features. While the reported consistency between GL-Net-derived and ground-truth-derived features is encouraging, the manuscript could more clearly articulate how classification consistency serves as a direct validation of segmentation quality. A brief clarification of this methodological rationale would strengthen the interpretability of the clinical validation.”

**Response:** We sincerely thank the reviewer for this insightful comment. We completely agree that explicitly stating the methodological rationale strengthens the logical link between our downstream clinical task and the upstream segmentation quality.

While geometric evaluation metrics (such as DSC and HD) are standard for assessing pixel-level spatial overlap, they do not necessarily reflect the clinical utility of the segmentation. In clinical practice, the key requirement of tumor segmentation is to accurately capture the structural and compositional features (e.g., the proportions of necrosis, enhancing, and non-enhancing subregions) that directly drive diagnostic decisions. Because the extraction of VASARI features heavily relies on the precise morphological boundaries and volume ratios of these subregions, achieving highly consistent downstream classification performance (and identical feature weight distributions) using GL-Net-generated masks compared to expert manual annotations (ground truth) directly proves that GL-Net accurately preserves clinically relevant morphological variations. This translates geometric accuracy into genuine clinical reliability.

To make this rationale clear to the readers, we have added an explanatory paragraph at the beginning of the "VASARI-based Feature Analysis" subsection in the revised manuscript:

*"While geometric metrics such as DSC and HD provide quantitative measures of spatial overlap and boundary distances, they do not fully capture the clinical utility of a segmentation model. In clinical workflows, the ultimate goal of tumor delineation is to extract reliable morphological and compositional metrics—such as the proportions of necrosis, enhancing, and non-enhancing tumor regions—that directly inform diagnosis and treatment planning. Therefore, downstream classification consistency serves as a crucial clinical validation of segmentation quality. If the segmentation masks generated by GL-Net yield VASARI features and subsequent GBM diagnostic performance that are statistically indistinguishable from those derived from expert manual annotations (ground truth), it directly demonstrates that GL-Net successfully preserves the clinically critical morphological variations and subregion proportions required for accurate diagnosis, thereby bridging the gap between pixel-level accuracy and clinical reliability."*

**5. Reviewer's Comment:** “Although the manuscript discusses clinical integration and workflow applicability, no external validation on independent real-world cohorts is provided. The current evaluation is limited to curated BraTS datasets. The claims regarding clinical applicability should be interpreted in light of this limitation, and a brief acknowledgment of the need for further prospective validation may improve balance.”

**Response:** We sincerely thank the reviewer for highlighting this critical limitation. We completely agree that performing well on curated public datasets, such as BraTS, does not automatically guarantee equivalent robustness in noisy, uncurated, real-world clinical environments. Our previous claims regarding clinical applicability were perhaps too optimistic without emphasizing this necessary caveat.

To provide a more balanced and scientifically rigorous perspective, we have tempered our claims regarding direct clinical applicability and explicitly acknowledged the lack of independent external validation as a primary limitation of our current study.

Specifically, we have substantially revised the final paragraph of the Conclusion section to read:

*"While GL-Net demonstrates strong segmentation accuracy and reliable downstream feature extraction, claims regarding its direct clinical applicability must be interpreted with caution. A primary limitation of the current study is the reliance on the highly curated BraTS datasets. Although multi-institutional, these standardized datasets may not fully capture the extreme heterogeneity, unpredictable artifacts, and diverse scanning protocols encountered in independent, real-world clinical* cohorts*. The absence of external validation on an independent real-world dataset means the model's operational robustness in everyday clinical practice remains to be definitively proven. Therefore, extensive external validation on independent clinical cohorts and subsequent prospective trials are essential next steps to rigorously evaluate the model's true generalizability..."*

**6. Reviewer's Comment:** “For full reproducibility, additional implementation details would be helpful. The manuscript does not specify the computational environment (e.g., GPU type), training time, inference *time*, or model parameter size. Including these details would improve transparency and practical reproducibility.”

**Response:** We sincerely thank the reviewer for this highly practical and constructive suggestion. We completely agree that detailing the computational environment, model size, and time efficiency is essential for ensuring full transparency and reproducibility, as well as for evaluating the feasibility of clinical deployment.

In response to your valuable feedback, we have updated the "Experimental Setup and Preprocessing" section of the revised manuscript to include all the requested implementation details. Specifically, we have added the following information:

*"All experiments were conducted on an Ubuntu operating system equipped with an Intel(R) Xeon(R) CPU E5-2678 v3 @ 2.50GHz, 128 GB of system RAM, and dual NVIDIA RTX 3090 Ti GPUs (24 GB VRAM each) …"*

**7. Reviewer's Comment:** “The manuscript refers to the ‘BraTS2021 test set,’ while also stating that five-fold cross-validation was performed. Since official test labels are not publicly available in BraTS *challenges*, the terminology should be clarified to avoid confusion regarding evaluation protocol.”

**Response:** We sincerely thank the reviewer for pointing out this terminological ambiguity. To avoid any confusion, we would like to clarify that because the official validation and test set ground truths are not publicly available, all evaluations in our study were conducted *exclusively* on the official BraTS training datasets. The "test sets" mentioned in our original manuscript were, in fact, the internal hold-out test folds generated during our five-fold cross-validation process on the official training data.

To ensure absolute clarity regarding our evaluation protocol, we have thoroughly revised the terminology throughout the manuscript:

1. In the **"Dataset and evaluation metrics"** section, we added an explicit statement: *"Since the ground truth labels for the official validation and test sets of BraTS2019 and BraTS2021 are withheld by the challenge organizers and are not publicly available, all models in this study were trained and evaluated exclusively on the official training datasets. To ensure a robust and fair evaluation, a five-fold cross-validation strategy was performed. Therefore, any reference to "test set" or "test results" in this paper specifically denotes the internal hold-out test folds partitioned during this cross-validation process, rather than the official unseen BraTS test sets."*

2. In the **"Comparative Experiments of GL-Net"** section and table captions (e.g., Table 5), we replaced the misleading phrase "BraTS test set" with **"internal hold-out test sets"** or **"internal test set"**.

Once again, we would like to express our deepest appreciation to the Editors and the Reviewers for their time, expertise, and highly constructive feedback. By addressing your insightful comments—specifically by incorporating rigorous statistical significance testing, clarifying mathematical formulations, providing detailed implementation metrics, standardizing our evaluation terminology, and offering a more balanced discussion on clinical applicability—we believe the methodological transparency, scientific rigor, and overall quality of our manuscript have been substantially elevated.

We hope that these comprehensive revisions satisfactorily address all your concerns and that the revised manuscript is now deemed suitable for publication in your journal. We remain fully available should you require any further clarifications or modifications.

Sincerely,

Huimin Lu
