# Supplementary material for: GL-Net: A knowledge-guided Gaussian-gated and layered refinement network for 3D MRI segmentation of brain gliomas
Source: PLoS One. 2026 Jun 22;21(6):e0351953. doi: 10.1371/journal.pone.0351953 (PMC13286167; doi:10.1371/journal.pone.0351953)
Supplement: Attachment — Submitted filename: Response_to_Reviewers_auresp_3.docx [file pone.0351953.s005.docx]

**Response to Reviewers**

Dear Editors and Reviewers,

We express our sincere gratitude to you and your colleagues for reviewing our submitted manuscript and providing valuable comments and suggestions. We highly appreciate these invaluable suggestions and believe they have played a crucial role in our research. We have carefully considered each of your suggestions, conducting thorough revisions to ensure the academic and methodological accuracy and credibility of our paper. Here, we sincerely respond to your review comments, hoping that our responses meet your expectations. Once again, we thank you for your valuable feedback and patience. Below are our responses to the specific comments:

**1. Reviewer's Comment:** “The authors have incorporated mean ± standard deviation and conducted paired t-tests across cross-validation folds, which is a welcome improvement. However, some concerns remain: The manuscript does not clarify whether the assumptions of the paired t-test (e.g., normality) were verified. Given the limited number of folds (n=5), the robustness of statistical testing may still be questioned.”

**Response:** We sincerely thank the reviewer for this insightful and constructive comment. We apologize for the lack of clarity regarding our statistical testing protocol and the rationale behind our cross-validation strategy. To address your concerns, we have clarified our methodology below and updated the manuscript accordingly.

1. We completely agree that strictly verifying the normality assumption is the best practice. Retrospectively, we conducted the Shapiro-Wilk test on the case-level differences. While most regions approximated normal distributions, a few exhibited slight skewness. To ensure absolute statistical rigor, we additionally performed the non-parametric Wilcoxon signed-rank test, which does not assume a normal distribution. The resulting p-values from the Wilcoxon test remain highly significant (p<0.01), perfectly consistent with the conclusions drawn from our original t-tests. We have updated the revised manuscript to explicitly describe this robust statistical procedure.

2. We deeply appreciate the reviewer’s concern regarding the number of folds. The choice of 5-fold cross-validation was a deliberate decision based on the bias-variance tradeoff. In our datasets (e.g., N=335 for BraTS 2019), increasing the number of folds (e.g., to 10-fold) would reduce the size of each validation set to only about 33 samples. Smaller validation sets are prone to high variance, meaning the performance metrics become overly sensitive to individual heterogeneous samples or noise, potentially leading to unreliable estimates of model generalization. A 5-fold CV ensures a more substantial validation set (n=67 per fold), providing a much more stable and representative estimate of the model. Furthermore, calculating 3D MRI configurations is computationally intensive; the 5-fold strategy optimally balances statistical stability and computational feasibility.

**2. Reviewer's Comment:** “The authors clarify that the results of comparative models were directly cited from the literature rather than re-implemented under identical experimental conditions. While this improves transparency, it raises concerns regarding fairness: Differences in preprocessing, training protocols, and data splits may significantly affect performance. Therefore, the comparison may not be strictly controlled. The authors should more explicitly acknowledge this limitation and moderate claims of superiority. Ideally, re-implementation under a unified framework would strengthen the validity of comparisons, though this may be left as future work.”

**Response:** We sincerely thank the reviewer for pointing out this critical caveat. We completely agree that comparing our results directly with those cited from the literature—without strictly controlling for preprocessing, exact cross-validation splits, and hardware/software environments—introduces potential biases and compromises the strict fairness of the comparison. we have made the following specific revisions to the manuscript:

1. We have significantly expanded the discussion in the “Comparative Experiments of GL-Net” section to explicitly warn readers that differences in data splits and pipelines may impact the performance metrics, and thus the comparisons should be interpreted as contextual benchmarks rather than strictly controlled head-to-head evaluations.

2. We have systematically reviewed the manuscript (especially the Abstract, Results, and Conclusion sections) and toned down definitive claims of “absolute superiority” (e.g., changing phrases like “outperforming several state-of-the-art algorithms” to “demonstrating highly competitive performance relative to reported benchmarks”).

3. As suggested, we have added a dedicated sentence in the “Conclusion” section stating that re-implementing these baseline models within a unified framework (e.g., standardized data splits and preprocessing pipelines) remains a crucial direction for our future work to establish more reliable comparisons.

**3. Reviewer's Comment:** “The authors have improved the clarity of variable definitions and provided a more structured explanation of the loss components. However: the final mathematical formulation of the combined loss function is still not explicitly presented in a concise equation. The relationship between the global Dice loss and the local boundary-restricted term would benefit from a clearer formal expression. Include an explicit final loss equation and ensure all symbols are consistently defined.”

**Response:** To address this issue, we have thoroughly revised the “Dynamic Weighted Loss Function for Edge Region Voxels” section. We have now decomposed the complex equation into three highly concise and explicit mathematical formulas:

1. The global Dice loss ($L_{Dice}$).

2. The boundary-restricted dynamic weighted cross-entropy loss ($L_{BRD}$).

3. The final combined loss equation expressed as a weighted sum ($L_{DiceBRD}=L_{Dice}+\lambda L_{BRD}$), which explicitly illustrates the relationship between the global and local terms.

Furthermore, immediately following these equations, we have provided a unified, bulleted list to define every mathematical symbol ($V,R,\mid R\mid,C,y_{ic},p_{ic},w_{i},\varepsilon,\lambda$) explicitly and consistently, leaving no room for ambiguity. We believe these modifications greatly enhance the mathematical readability and exactness of our proposed method. Please see the revised manuscript for these specific changes.

**4. Reviewer's Comment:** “The authors provide a more detailed explanation of using VASARI-based classification consistency as a proxy for segmentation quality. This clarification is helpful. However: the argument remains somewhat overstated. The claim that downstream consistency “directly proves” segmentation quality is too strong. Rephrase this argument to reflect that downstream consistency provides supporting or indirect evidence, rather than definitive proof.”

**Response:** We sincerely appreciate the reviewer’s careful reading and valid critique regarding the precision of our scientific claims. We completely agree that claiming downstream classification consistency “directly demonstrates” segmentation quality is overly absolute. While downstream task performance highlights the clinical utility of the generated masks, it is ultimately an indirect measure and should be properly framed as complementary evidence alongside geometric metrics like DSC and HD, rather than definitive proof of pixel-perfect segmentation. In accordance with your excellent suggestion, we have systematically reviewed the manuscript and meticulously toned down these claims, particularly in the “Abstract”, “VASARI-based Feature Analysis”, and “Conclusion” sections. We have rephrased the relevant sentences to explicitly state that downstream consistency “provides valuable complementary and indirect evidence” of the model’s reliability and clinical utility. We believe these revisions significantly enhance the scientific rigor and objectivity of our manuscript.

**5. Reviewer's Comment:** “The authors have appropriately acknowledged the limitation regarding the lack of external validation and have moderated their claims. Well, this is a strong improvement, and the revised discussion is now more balanced and scientifically appropriate. Also, the addition of hardware specifications is appreciated. However, important details are still missing: Model parameter size, Training time, Inference time. Include these details to ensure full reproducibility and to allow readers to assess computational feasibility.”

**Response:** We sincerely thank the reviewer for the highly encouraging feedback and for recognizing the improvements in our revised manuscript. We also appreciate you emphasizing the importance of computational details.

We apologize if these specific metrics were not prominent enough in our previous revision, which might have caused them to be overlooked. We are pleased to clarify that we have indeed included these exact details at the end of the **“Experimental Setup and Preprocessing”** section. To ensure absolute clarity, we summarize them here:

**1. Model Parameter Size:** The proposed GL-Net has a total parameter count of approximately **35.2 M**;

**2. Training Time:** Under our hardware configuration (dual NVIDIA RTX 3090 Ti GPUs), the total training time for 100 epochs was approximately **14 hours**.

**3. Inference Time:** During the testing phase, the average inference time per patient volume (a full 3D MRI scan) was strictly around **4.2 seconds** (approx. 27 ms per slice).

We hope that these comprehensive revisions satisfactorily address all your concerns and that the revised manuscript is now deemed suitable for publication in your journal

Sincerely,

Huimin Lu
