# Supplementary material for: GL-Net: A knowledge-guided Gaussian-gated and layered refinement network for 3D MRI segmentation of brain gliomas
Source: PLoS One. 2026 Jun 22;21(6):e0351953. doi: 10.1371/journal.pone.0351953 (PMC13286167; doi:10.1371/journal.pone.0351953)
Supplement: Attachment — Submitted filename: Response_to_Reviewers_auresp_4.docx [file pone.0351953.s006.docx]

**Response to Reviewers**

Dear Editors and Reviewers,

We express our sincere gratitude to you and your colleagues for reviewing our submitted manuscript and providing valuable comments and suggestions. We highly appreciate these invaluable suggestions and believe they have played a crucial role in our research. We have carefully considered each of your suggestions, conducting thorough revisions to ensure the academic and methodological accuracy and credibility of our paper. Here, we sincerely respond to your review comments, hoping that our responses meet your expectations. Once again, we thank you for your valuable feedback and patience. Below are our responses to the specific comments:

**1. Reviewer's Comment:** “The claim of superiority over existing methods should be softened, as comparisons were not made in a strictly controlled environment. Please revise it to reflect competitive performance. The author is using very dangerous wording. "Comparison is not strictly controlled..." is incomprehensible to an experimenter.”

**Response:** We sincerely thank the reviewer for this critical and highly constructive feedback. We completely agree that claiming absolute superiority is scientifically inappropriate when comparing our results with metrics directly cited from other publications, given the inevitable differences in data splits, preprocessing pipelines, and evaluation frameworks. We also deeply appreciate the reviewer pointing out the inappropriate and potentially misleading phrase "Comparison is not strictly controlled". To address this, we have thoroughly revised the manuscript. Specifically, we have:

1. Removed all variations of the phrase "not strictly controlled" and replaced them with standard academic terminology (e.g., "indirect comparison," "different evaluation frameworks").

2. Softened words like "superior," "outperforming," and "best" to "highly competitive," "comparable to state-of-the-art," and "demonstrates robust performance" throughout the Abstract, Results, and Conclusion sections.

3. 3.	Clarified that our results show the model's strong potential and competitiveness, while explicitly acknowledging that a definitive head-to-head evaluation would require re-implementing all models within a unified framework (such as nnU-Net).

**2. Reviewer's Comment:** “Clinical claims should be softened. In the absence of external validation, expressions such as "clinical utility" should be revised to indicate potential clinical relevance.”

**Response:** We sincerely thank the reviewer for pointing this out. We fully agree that without validation on an independent, external clinical dataset, claiming "clinical utility" or "direct clinical applicability" is scientifically premature and overstates the current developmental stage of our model.

To ensure scientific rigor and adhere to standard reporting practices in medical image analysis, we have thoroughly reviewed the manuscript and softened all relevant clinical claims. Specifically, we have replaced terms such as "clinical utility" and "practical utility" with more objective and cautious expressions, including "potential clinical relevance," "translational potential," and "clinical potential." Furthermore, we have explicitly highlighted the critical need for future external validation on independent cohorts before any true clinical utility can be established. We believe these revisions accurately reflect the current limitations and the true scope of our work.

**3. Reviewer's Comment:** “p-values reported as "0.00" should be corrected to a standard format (e.g., p < 0.01).”

**Response:** We sincerely thank the reviewer for pointing out this formatting issue. We completely agree that reporting a p-value exactly as "0.00" is statistically inaccurate. Following your suggestion, we have carefully reviewed the entire manuscript, including all relevant texts and tables (Tables 3, 4, and 5). We have corrected all instances of "0.00" to the standard format "< 0.01". Accordingly, we have also updated the table footnotes to reflect these changes. We appreciate your rigorous attention to detail, which has helped us improve the statistical reporting standard of our paper.

We hope that these comprehensive revisions satisfactorily address all your concerns and that the revised manuscript is now deemed suitable for publication in your journal

Sincerely,

Huimin Lu
