# Supplementary material for: GL-Net: A knowledge-guided Gaussian-gated and layered refinement network for 3D MRI segmentation of brain gliomas
Source: PLoS One. 2026 Jun 22;21(6):e0351953. doi: 10.1371/journal.pone.0351953 (PMC13286167; doi:10.1371/journal.pone.0351953)
Supplement: Peer review history

# Peer review: Decision Letter 0

**DOI:** 10.1371/journal.pone.0351953.r001

**Authors:** Mario Tortora

16 Oct 2025

PONE-D-25-43663

GL-Net: Gaussian-Gated and Layered Refinement Algorithm for MRI Segmentation of Brain Gliomas

PLOS ONE

Dear Dr. Lu,

Thank you for submitting your manuscript to PLOS ONE. After careful consideration, we feel that it has merit but does not fully meet PLOS ONE’s publication criteria as it currently stands. Therefore, we invite you to submit a revised version of the manuscript that addresses the points raised during the review process.

Please submit your revised manuscript by Nov 30 2025 11:59PM. If you will need more time than this to complete your revisions, please reply to this message or contact the journal office at plosone@plos.org. When you're ready to submit your revision, log on to https://www.editorialmanager.com/pone/ and select the 'Submissions Needing Revision' folder to locate your manuscript file.

Please include the following items when submitting your revised manuscript:

- A rebuttal letter that responds to each point raised by the academic editor and reviewer(s). You should upload this letter as a separate file labeled 'Response to Reviewers'.
- A marked-up copy of your manuscript that highlights changes made to the original version. You should upload this as a separate file labeled 'Revised Manuscript with Track Changes'.
- An unmarked version of your revised paper without tracked changes. You should upload this as a separate file labeled 'Manuscript'.

If you would like to make changes to your financial disclosure, please include your updated statement in your cover letter. Guidelines for resubmitting your figure files are available below the reviewer comments at the end of this letter.

If applicable, we recommend that you deposit your laboratory protocols in protocols.io to enhance the reproducibility of your results. Protocols.io assigns your protocol its own identifier (DOI) so that it can be cited independently in the future. For instructions see: https://journals.plos.org/plosone/s/submission-guidelines#loc-laboratory-protocols. Additionally, PLOS ONE offers an option for publishing peer-reviewed Lab Protocol articles, which describe protocols hosted on protocols.io. Read more information on sharing protocols at https://plos.org/protocols?utm_medium=editorial-email&utm_source=authorletters&utm_campaign=protocols.

We look forward to receiving your revised manuscript.

Kind regards,

Mario Tortora, Pd.D.s, M.D.

Academic Editor

PLOS ONE

**Journal Requirements:**

1. When submitting your revision, we need you to address these additional requirements.

Please ensure that your manuscript meets PLOS ONE's style requirements, including those for file naming. The PLOS ONE style templates can be found at

https://journals.plos.org/plosone/s/file?id=wjVg/PLOSOne_formatting_sample_main_body.pdf and

https://journals.plos.org/plosone/s/file?id=ba62/PLOSOne_formatting_sample_title_authors_affiliations.pdf

2. Please note that PLOS One has specific guidelines on code sharing for submissions in which author-generated code underpins the findings in the manuscript. In these cases, we expect all author-generated code to be made available without restrictions upon publication of the work. Please review our guidelines at https://journals.plos.org/plosone/s/materials-and-software-sharing#loc-sharing-code and ensure that your code is shared in a way that follows best practice and facilitates reproducibility and reuse.

3. Thank you for stating in your Funding Statement:

This research is supported by the Development Special Project of Jilin Provincial Development and Reform Commission in 2023 (No. 2023C042-6).

Please provide an amended statement that declares *all* the funding or sources of support (whether external or internal to your organization) received during this study, as detailed online in our guide for authors at http://journals.plos.org/plosone/s/submit-now.  Please also include the statement “There was no additional external funding received for this study.” in your updated Funding Statement.

Please include your amended Funding Statement within your cover letter. We will change the online submission form on your behalf.

4. Thank you for uploading your study's underlying data set. Unfortunately, the repository you have noted in your Data Availability statement does not qualify as an acceptable data repository according to PLOS's standards.

At this time, please upload the minimal data set necessary to replicate your study's findings to a stable, public repository (such as figshare or Dryad) and provide us with the relevant URLs, DOIs, or accession numbers that may be used to access these data. For a list of recommended repositories and additional information on PLOS standards for data deposition, please see https://journals.plos.org/plosone/s/recommended-repositories.

5. If the reviewer comments include a recommendation to cite specific previously published works, please review and evaluate these publications to determine whether they are relevant and should be cited. There is no requirement to cite these works unless the editor has indicated otherwise.

**Additional Editor Comments:**

This manuscript presents a technically interesting and potentially impactful approach to 3D glioblastoma segmentation using a hybrid transformer–CNN architecture. However, its clinical positioning remains underdeveloped. The authors are strongly encouraged to better contextualize their model within current clinical practice by integrating relevant literature and aligning their discussion with existing radiological evaluation frameworks. Further comparison with prior models will also be necessary to objectively assess the proposed method’s contribution to the field.

Title:

The title is clear and informative, but could be further strengthened by explicitly highlighting the methodological innovation introduced in the paper, to better convey its novelty and relevance compared to existing literature.

Abstract:

The abstract is well written and provides a coherent summary of the study. However, it would benefit from the inclusion of more specific performance results and a brief mention of the potential clinical implications of the proposed method, in order to emphasize its real-world utility.

Introduction:

The introduction is well structured and successfully establishes the clinical relevance of accurate glioblastoma segmentation. That said, to improve scientific depth and contextual clarity, the background section should be expanded to include more details on current radiological assessment frameworks commonly used in clinical practice for glioma characterization. Including references to standardized descriptors would strengthen the clinical foundation of the work and better justify the need for automated approaches.

Materials and Methods:

The methodology is described in sufficient detail. However, clarification is needed on whether the dataset reflects adequate clinical and technical heterogeneity (e.g., use of different MRI scanners and acquisition protocols), to ensure that the proposed model generalizes well across varying real-world conditions.

Statistical Analysis:

The statistical analysis is sound and appropriately rigorous. Nonetheless, the authors should discuss potential limitations related to dataset size, class imbalance, or selection bias, and how these factors may have influenced the results.

Results:

The results are clearly presented. However, a more direct and quantitative comparison with existing models is encouraged—ideally through a summary table—to help readers appreciate the degree of improvement introduced by the proposed method.

Discussion:

The discussion effectively highlights the main contributions of the work, but it would benefit from a more critical integration of recent literature, particularly studies that bridge conventional MRI evaluation and computational methods. Including references to structured radiological reporting tools and lexicons currently used in glioma assessment would enhance the discussion and help demonstrate how the proposed model might complement existing clinical workflows.

Conclusion:

The conclusions are consistent with the reported results. However, they should more explicitly acknowledge that implementation in clinical practice requires further validation, particularly in relation to how the model correlates with standard radiological evaluation criteria. The authors should stress that clinical translation depends on how well the algorithm integrates with expert visual assessment and standardized MRI descriptors.

Figures, Tables, and Diagrams:

Figures and tables are high quality, well-designed, and clearly aid in data interpretation. It may be useful to add a practical example or visual workflow illustrating how the model could be integrated into a clinical imaging pipeline.

References:

The references are appropriate and up to date. However, to further strengthen the clinical relevance of the work, it is recommended that the authors include recent studies that explore the use of structured MRI descriptors and visual rating systems for glioma grading and molecular status prediction, especially those based on conventional imaging protocols.

[Note: HTML markup is below. Please do not edit.]

Reviewers' comments:

Reviewer's Responses to Questions

**Comments to the Author**

1. Is the manuscript technically sound, and do the data support the conclusions?

The manuscript must describe a technically sound piece of scientific research with data that supports the conclusions. Experiments must have been conducted rigorously, with appropriate controls, replication, and sample sizes. The conclusions must be drawn appropriately based on the data presented.

Reviewer #1: Yes

**********

2. Has the statistical analysis been performed appropriately and rigorously?

Reviewer #1: Yes

**********

3. Have the authors made all data underlying the findings in their manuscript fully available?

The PLOS Data policy requires authors to make all data underlying the findings described in their manuscript fully available without restriction, with rare exception (please refer to the Data Availability Statement in the manuscript PDF file). The data should be provided as part of the manuscript or its supporting information, or deposited to a public repository. For example, in addition to summary statistics, the data points behind means, medians and variance measures should be available. If there are restrictions on publicly sharing data—e.g. participant privacy or use of data from a third party—those must be specified.

Reviewer #1: Yes

**********

4. Is the manuscript presented in an intelligible fashion and written in standard English?

PLOS ONE does not copyedit accepted manuscripts, so the language in submitted articles must be clear, correct, and unambiguous. Any typographical or grammatical errors should be corrected at revision, so please note any specific errors here.

Reviewer #1: Yes

**********

5. Review Comments to the Author

Please use the space provided to explain your answers to the questions above. You may also include additional comments for the author, including concerns about dual publication, research ethics, or publication ethics. (Please upload your review as an attachment if it exceeds 20,000 characters)

**Reviewer #1:**   Clear and concise title.

The well-written and comprehensive abstract describes the submitted scientific work in its entirety, providing references to the scientific background and clarifying the study hypotheses.

The well-structured introduction. Glioblastoma is a highly malignant brain tumor, and accurate lesion segmentation on imaging plays a crucial role in the diagnosis, treatment, and follow-up of glioblastoma. The challenge of this work is to propose a 3D segmentation method for glioblastoma MRI, GL-Net. This method employs an improved hybrid architecture that integrates a transformer and a CNN (T-CNN), integrating knowledge-based features into traditional data-driven approaches.

The materials and methods are well described.

The statistical analysis is thorough and rigorous.

The results are presented clearly and comprehensively.

In the discussion, they are analyzed point by point and compared with other similar articles recently published on the topic, highlighting the strengths and major innovations emerging from the submitted paper.

The conclusions are supported by the results and appear interesting.

Detailed and appropriate tables, graphs, and diagrams make understanding easier and faster.

Well-curated images.

Appropriate bibliographic references are provided, including references to the most recent major scientific articles published on the topic.

I suggest including in the discussion or in the conclusion that the proposed method for the automatic 3D segmentation of brain gliomas must be implemented in daily clinical practice and must be correlated with the clinical radiological assessment based on the identification of various MRI characteristics, first and foremost those of the conventional MRI examination, such as those codified by the so-called VASARI lexicon

(Below I report some bibliographical references on the topic: “VASARI 2.0: a new updated MRI VASARI lexicon to predict grading and IDH status in brain glioma. Negro A, Gemini L, Tortora M, Pace G, Iaccarino R, Marchese M, Elefante A, Tortora F, D'Agostino V; members of ODM Multidisciplinary Neuro-Oncology Group. Front Oncol. 2024 Dec 23;14:1449982. doi: 10.3389/fonc.2024.1449982. eCollection 2024. PMID: 39763601; “Vasari Scoring System in Discerning between Different Degrees of Glioma and IDH Status Prediction: A Possible Machine Learning Application? Gemini L, Tortora M, Giordano P, Prudente ME, Villa A, Vargas O, Giugliano MF, Somma F, Marchello G, Chiaramonte C, Gaetano M, Frio F, Di Giorgio E, D'Avino A, Tortora F, D'Agostino V, Negro A. J Imaging. 2023 Mar 24;9(4):75. doi: 10.3390/jimaging9040075. PMID: 37103226”).

**********

6. PLOS authors have the option to publish the peer review history of their article (what does this mean?). If published, this will include your full peer review and any attached files.

If you choose “no”, your identity will remain anonymous but your review may still be made public.

**Do you want your identity to be public for this peer review?**   For information about this choice, including consent withdrawal, please see our Privacy Policy.

Reviewer #1: **Yes:**   Alberto Negro

**********

[NOTE: If reviewer comments were submitted as an attachment file, they will be attached to this email and accessible via the submission site. Please log into your account, locate the manuscript record, and check for the action link "View Attachments". If this link does not appear, there are no attachment files.]

While revising your submission, please upload your figure files to the Preflight Analysis and Conversion Engine (PACE) digital diagnostic tool, https://pacev2.apexcovantage.com/. PACE helps ensure that figures meet PLOS requirements. To use PACE, you must first register as a user. Registration is free. Then, login and navigate to the UPLOAD tab, where you will find detailed instructions on how to use the tool. If you encounter any issues or have any questions when using PACE, please email PLOS at figures@plos.org. Please note that Supporting Information files do not need this step.

# Author comment: Author response to Decision Letter 1

**DOI:** 10.1371/journal.pone.0351953.r002

17 Nov 2025

Dear Editors and Reviewers,

We express our sincere gratitude to you and your colleagues for reviewing our submitted manuscript and providing valuable comments and suggestions. We highly appreciate these invaluable suggestions and believe they have played a crucial role in our research. We have carefully considered each of your suggestions, conducting thorough revisions to ensure the academic and methodological accuracy and credibility of our paper. Here, we sincerely respond to your review comments, hoping that our responses meet your expectations. Once again, we thank you for your valuable feedback and patience. Below are our responses to the specific comments:

Additional Editor Comments:

“This manuscript presents a technically interesting and potentially impactful approach to 3D glioblastoma segmentation using a hybrid transformer–CNN architecture. However, its clinical positioning remains underdeveloped. The authors are strongly encouraged to better contextualize their model within current clinical practice by integrating relevant literature and aligning their discussion with existing radiological evaluation frameworks. Further comparison with prior models will also be necessary to objectively assess the proposed method’s contribution to the field.

Title:

The title is clear and informative, but could be further strengthened by explicitly highlighting the methodological innovation introduced in the paper, to better convey its novelty and relevance compared to existing literature.

Abstract:

The abstract is well written and provides a coherent summary of the study. However, it would benefit from the inclusion of more specific performance results and a brief mention of the potential clinical implications of the proposed method, in order to emphasize its real-world utility.

Introduction:

The introduction is well structured and successfully establishes the clinical relevance of accurate glioblastoma segmentation. That said, to improve scientific depth and contextual clarity, the background section should be expanded to include more details on current radiological assessment frameworks commonly used in clinical practice for glioma characterization. Including references to standardized descriptors would strengthen the clinical foundation of the work and better justify the need for automated approaches.

Materials and Methods:

The methodology is described in sufficient detail. However, clarification is needed on whether the dataset reflects adequate clinical and technical heterogeneity (e.g., use of different MRI scanners and acquisition protocols), to ensure that the proposed model generalizes well across varying real-world conditions.

Statistical Analysis:

The statistical analysis is sound and appropriately rigorous. Nonetheless, the authors should discuss potential limitations related to dataset size, class imbalance, or selection bias, and how these factors may have influenced the results.

Results:

The results are clearly presented. However, a more direct and quantitative comparison with existing models is encouraged—ideally through a summary table—to help readers appreciate the degree of improvement introduced by the proposed method.

Discussion:

The discussion effectively highlights the main contributions of the work, but it would benefit from a more critical integration of recent literature, particularly studies that bridge conventional MRI evaluation and computational methods. Including references to structured radiological reporting tools and lexicons currently used in glioma assessment would enhance the discussion and help demonstrate how the proposed model might complement existing clinical workflows.

Conclusion:

The conclusions are consistent with the reported results. However, they should more explicitly acknowledge that implementation in clinical practice requires further validation, particularly in relation to how the model correlates with standard radiological evaluation criteria. The authors should stress that clinical translation depends on how well the algorithm integrates with expert visual assessment and standardized MRI descriptors.”

Reply to Additional Editor:

It is our great honor that our work has been recognized by the editors, and we sincerely appreciate the constructive comments provided. Below, we provide our responses to the suggestions for each section.

Response regarding the title:

To better highlight the methodological innovation and emphasize the novelty of our approach, we have revised the title to explicitly reflect the integration of prior knowledge and the key technical modules (Gaussian-Gated and Layered Refinement). The revised title now reads: “GL-Net: A Knowledge-Guided Gaussian-Gated and Layered Refinement Network for 3D MRI Segmentation of Brain Gliomas.” This modification better conveys the methodological contribution of our work while keeping the title concise and informative.

Response regarding the abstract:

To better emphasize the real-world applicability and performance of our proposed method, we have revised the abstract to include the quantitative analysis results based on VASARI features extracted from the segmentation outputs. We also briefly highlighted the potential clinical significance of GL-Net in supporting accurate glioma diagnosis and treatment planning. The revised abstract now provides clearer evidence of the model’s effectiveness and clinical relevance.

Response regarding Materials and Methods:

In the revised section, we have added a detailed description of the datasets used, including data sources, imaging device manufacturers, magnetic field strength ranges, and relevant imaging protocols. These additions ensure that the proposed model can be well generalized across diverse real-world conditions.

Response regarding statistical analysis:

This is an excellent suggestion. The issues raised—such as dataset size, class imbalance, and selection bias—are indeed among the key challenges that our work aims to address. Following the editors’ recommendation, we have incorporated additional content in the Results section to specifically discuss these aspects.

Response regarding the Results:

We have optimized the presentation of the summary tables and added new figures to illustrate the results. We believe these additions will help readers more intuitively understand the extent of improvements achieved by the proposed method.

Response regarding the Discussion and Conclusion:

In these two sections, we have added content on the quantitative VASARI evaluation of segmentation masks produced by our model compared with the gold-standard masks delineated by clinicians. The results demonstrate that the proposed model holds substantial potential and utility in supporting clinical practice and subsequent research.

Response regarding Figures, Tables, and Diagrams:

In the Methods section, we have added a workflow diagram to illustrate how the model can be integrated into the clinical workflow.

Response regarding References:

We have added new references in the manuscript concerning the use of structured MRI descriptors and the VASARI visual assessment system for glioma grading.

Reviewer #1: I suggest including in the discussion or in the conclusion that the proposed method for the automatic 3D segmentation of brain gliomas must be implemented in daily clinical practice and must be correlated with the clinical radiological assessment based on the identification of various MRI characteristics, first and foremost those of the conventional MRI examination, such as those codified by the so-called VASARI lexicon.

Reply to Reviewer 1:

Thank you very much for your careful review and approval of our paper. The suggestions and viewpoints you provided have played a crucial guiding role in our research. Here are our responses to specific comments:

Following your suggestion, we conducted additional experiments to further evaluate the clinical applicability of our method. Specifically, we extracted F4–F7 structural imaging features based on the VASARI scoring system from both GL-Net segmentation outputs and the gold-standard masks. This allowed us to assess the consistency and reliability of the model’s segmentation results in the context of GBM clinical characterization, and to verify that the model’s support for clinical diagnosis is comparable to manual annotation.

The results showed that, after extracting VASARI structural imaging features from both GL-Net and BraTS gold-standard masks, we built GBM classification models and compared the discriminative performance of the two feature sets. The model based on gold-standard masks achieved an AUC of 0.954, ACC of 0.940, and F1 score of 0.962, whereas the model based on GL-Net outputs achieved an AUC of 0.949, ACC of 0.925, and F1 score of 0.952, demonstrating a high degree of consistency across all metrics. DeLong’s test further indicated that the difference in AUC between the two models was not statistically significant (p = 0.9996).

Additionally, we visualized the feature weights of the logistic regression models (Figure X) and observed that the weight distribution patterns were nearly identical between the two models. This further confirms that the structural imaging features derived from GL-Net segmentations contribute to GBM classification in a manner highly consistent with manual annotations.

In summary, the GL-Net segmentation masks provide stable and reliable structural imaging features for GBM clinical feature extraction and diagnostic discrimination, demonstrating comparable performance to manual gold-standard masks. These findings indicate that GL-Net has strong potential for integration into daily clinical practice, supporting radiological assessment based on MRI characteristics, including those codified by the VASARI lexicon, and facilitating accurate and efficient clinical decision-making.

Sincerely,

Huimin Lu

Attachment Submitted filename: Response to Reviewers.docx

# Peer review: Decision Letter 1

**DOI:** 10.1371/journal.pone.0351953.r003

**Authors:** Taikyeong Jeong

19 Feb 2026

PONE-D-25-43663R1

GL-Net: A Knowledge-Guided Gaussian-Gated and Layered Refinement Network for 3D MRI Segmentation of Brain Gliomas

PLOS One

Dear Dr. Lu,

Thank you for submitting your manuscript to PLOS ONE. After careful consideration, we feel that it has merit but does not fully meet PLOS ONE’s publication criteria as it currently stands. Therefore, we invite you to submit a revised version of the manuscript that addresses the points raised during the review process.

==============================

Although the manuscript presents a technically sound and well-structured study, several issues may warrant minor revision.

While the manuscript reports improvements in DSC and HD metrics, no statistical significance testing is provided to support these performance gains. Given that some improvements are relatively modest in magnitude, it would be important to demonstrate whether the reported differences are statistically significant across folds. Reporting mean ± standard deviation and including appropriate paired statistical tests (e.g., paired t-test or Wilcoxon signed-rank test) would strengthen the robustness of the conclusions.

Tables 1 and 2 compare GL-Net with several existing models. However, it is not clearly stated whether these models were re-implemented under identical preprocessing and cross-validation settings, or whether the results were directly cited from the original publications. This distinction is important for assessing fairness and reproducibility. Explicit clarification of the comparison protocol would improve methodological transparency.

The mathematical formulation of the DiceBRD loss function requires clearer explanation. In particular, the relationship between the ROI-based region restriction and the combined Dice and weighted cross-entropy terms could be more explicitly described. Additionally, certain variable definitions appear repetitive or ambiguous. A more precise and structured presentation of the loss formulation would enhance technical clarity.

The manuscript evaluates segmentation performance indirectly through downstream GBM classification using selected VASARI features. While the reported consistency between GL-Net-derived and ground-truth-derived features is encouraging, the manuscript could more clearly articulate how classification consistency serves as a direct validation of segmentation quality. A brief clarification of this methodological rationale would strengthen the interpretability of the clinical validation.

Although the manuscript discusses clinical integration and workflow applicability, no external validation on independent real-world cohorts is provided. The current evaluation is limited to curated BraTS datasets. The claims regarding clinical applicability should be interpreted in light of this limitation, and a brief acknowledgment of the need for further prospective validation may improve balance.

For full reproducibility, additional implementation details would be helpful. The manuscript does not specify the computational environment (e.g., GPU type), training time, inference time, or model parameter size. Including these details would improve transparency and practical reproducibility.

The manuscript refers to the “BraTS2021 test set,” while also stating that five-fold cross-validation was performed. Since official test labels are not publicly available in BraTS challenges, the terminology should be clarified to avoid confusion regarding evaluation protocol.

The manuscript demonstrates solid technical contribution and meaningful clinical contextualization. The issues raised above do not undermine the validity of the study but relate primarily to clarity, statistical rigor, and methodological transparency.

==============================

Please submit your revised manuscript by Apr 05 2026 11:59PM. If you will need more time than this to complete your revisions, please reply to this message or contact the journal office at plosone@plos.org. When you're ready to submit your revision, log on to https://www.editorialmanager.com/pone/ and select the 'Submissions Needing Revision' folder to locate your manuscript file.

Please include the following items when submitting your revised manuscript:

- A letter that responds to each point raised by the academic editor and reviewer(s). You should upload this letter as a separate file labeled 'Response to Reviewers'.
- A marked-up copy of your manuscript that highlights changes made to the original version. You should upload this as a separate file labeled 'Revised Manuscript with Track Changes'.
- An unmarked version of your revised paper without tracked changes. You should upload this as a separate file labeled 'Manuscript'.

-->If you would like to make changes to your financial disclosure, please include your updated statement in your cover letter. Guidelines for resubmitting your figure files are available below the reviewer comments at the end of this letter.

If applicable, we recommend that you deposit your laboratory protocols in protocols.io to enhance the reproducibility of your results. Protocols.io assigns your protocol its own identifier (DOI) so that it can be cited independently in the future. For instructions see: https://journals.plos.org/plosone/s/submission-guidelines#loc-laboratory-protocols. Additionally, PLOS ONE offers an option for publishing peer-reviewed Lab Protocol articles, which describe protocols hosted on protocols.io. Read more information on sharing protocols at https://plos.org/protocols?utm_medium=editorial-email&utm_source=authorletters&utm_campaign=protocols.

We look forward to receiving your revised manuscript.

Kind regards,

Taikyeong Ted Jeong, Ph.D.

Academic Editor

PLOS One

**Journal Requirements:**

1. If the reviewer comments include a recommendation to cite specific previously published works, please review and evaluate these publications to determine whether they are relevant and should be cited. There is no requirement to cite these works unless the editor has indicated otherwise.

2. Please review your reference list to ensure that it is complete and correct. If you have cited papers that have been retracted, please include the rationale for doing so in the manuscript text, or remove these references and replace them with relevant current references. Any changes to the reference list should be mentioned in the rebuttal letter that accompanies your revised manuscript. If you need to cite a retracted article, indicate the article’s retracted status in the References list and also include a citation and full reference for the retraction notice.

**Additional Editor Comments:**

Although the manuscript presents a technically sound and well-structured study, several issues may warrant minor revision.

While the manuscript reports improvements in DSC and HD metrics, no statistical significance testing is provided to support these performance gains. Given that some improvements are relatively modest in magnitude, it would be important to demonstrate whether the reported differences are statistically significant across folds. Reporting mean ± standard deviation and including appropriate paired statistical tests (e.g., paired t-test or Wilcoxon signed-rank test) would strengthen the robustness of the conclusions.

Tables 1 and 2 compare GL-Net with several existing models. However, it is not clearly stated whether these models were re-implemented under identical preprocessing and cross-validation settings, or whether the results were directly cited from the original publications. This distinction is important for assessing fairness and reproducibility. Explicit clarification of the comparison protocol would improve methodological transparency.

The mathematical formulation of the DiceBRD loss function requires clearer explanation. In particular, the relationship between the ROI-based region restriction and the combined Dice and weighted cross-entropy terms could be more explicitly described. Additionally, certain variable definitions appear repetitive or ambiguous. A more precise and structured presentation of the loss formulation would enhance technical clarity.

The manuscript evaluates segmentation performance indirectly through downstream GBM classification using selected VASARI features. While the reported consistency between GL-Net-derived and ground-truth-derived features is encouraging, the manuscript could more clearly articulate how classification consistency serves as a direct validation of segmentation quality. A brief clarification of this methodological rationale would strengthen the interpretability of the clinical validation.

Although the manuscript discusses clinical integration and workflow applicability, no external validation on independent real-world cohorts is provided. The current evaluation is limited to curated BraTS datasets. The claims regarding clinical applicability should be interpreted in light of this limitation, and a brief acknowledgment of the need for further prospective validation may improve balance.

For full reproducibility, additional implementation details would be helpful. The manuscript does not specify the computational environment (e.g., GPU type), training time, inference time, or model parameter size. Including these details would improve transparency and practical reproducibility.

The manuscript refers to the “BraTS2021 test set,” while also stating that five-fold cross-validation was performed. Since official test labels are not publicly available in BraTS challenges, the terminology should be clarified to avoid confusion regarding evaluation protocol.

The manuscript demonstrates solid technical contribution and meaningful clinical contextualization. The issues raised above do not undermine the validity of the study but relate primarily to clarity, statistical rigor, and methodological transparency.

[Note: HTML markup is below. Please do not edit.]

Reviewers' comments:

Reviewer's Responses to Questions

**Comments to the Author**

1. If the authors have adequately addressed your comments raised in a previous round of review and you feel that this manuscript is now acceptable for publication, you may indicate that here to bypass the “Comments to the Author” section, enter your conflict of interest statement in the “Confidential to Editor” section, and submit your "Accept" recommendation.

Reviewer #2: All comments have been addressed

Reviewer #3: All comments have been addressed

Reviewer #4: (No Response)

**********

2. Is the manuscript technically sound, and do the data support the conclusions?

The manuscript must describe a technically sound piece of scientific research with data that supports the conclusions. Experiments must have been conducted rigorously, with appropriate controls, replication, and sample sizes. The conclusions must be drawn appropriately based on the data presented.

Reviewer #2: Yes

Reviewer #3: Yes

Reviewer #4: Partly

**********

3. Has the statistical analysis been performed appropriately and rigorously?

Reviewer #2: Yes

Reviewer #3: Yes

Reviewer #4: Yes

**********

4. Have the authors made all data underlying the findings in their manuscript fully available?

The PLOS Data policy requires authors to make all data underlying the findings described in their manuscript fully available without restriction, with rare exception (please refer to the Data Availability Statement in the manuscript PDF file). The data should be provided as part of the manuscript or its supporting information, or deposited to a public repository. For example, in addition to summary statistics, the data points behind means, medians and variance measures should be available. If there are restrictions on publicly sharing data—e.g. participant privacy or use of data from a third party—those must be specified.

Reviewer #2: Yes

Reviewer #3: Yes

Reviewer #4: Yes

**********

5. Is the manuscript presented in an intelligible fashion and written in standard English?

PLOS ONE does not copyedit accepted manuscripts, so the language in submitted articles must be clear, correct, and unambiguous. Any typographical or grammatical errors should be corrected at revision, so please note any specific errors here.

Reviewer #2: Yes

Reviewer #3: Yes

Reviewer #4: Yes

**********

6. Review Comments to the Author

Please use the space provided to explain your answers to the questions above. You may also include additional comments for the author, including concerns about dual publication, research ethics, or publication ethics. (Please upload your review as an attachment if it exceeds 20,000 characters)

**Reviewer #2:**   The author have already met the requirements above, and I suppose the essay should be accepted. Furthermore, I believe that this essay involves reasonable innovations.

**Reviewer #3:**   The manuscript is scientifically rigorous and suitable for PLOS ONE, with strong potential impact in medical image analysis. However, revisions are needed to address benchmarking limitations, enhance reproducibility (e.g., release code), and provide more cautious interpretation of results. Resubmit with clarified comparisons to official BraTS metrics where possible, and consider external validation on a small held-out cohort if feasible.

**Reviewer #4:**   The authors answered satisfactory to previous Referee report, however they do not take into account the role of the environmental noise. See the attached report.

**********

7. PLOS authors have the option to publish the peer review history of their article (what does this mean?). If published, this will include your full peer review and any attached files.

If you choose “no”, your identity will remain anonymous but your review may still be made public.

**Do you want your identity to be public for this peer review?**   For information about this choice, including consent withdrawal, please see our Privacy Policy.

Reviewer #2: No

Reviewer #3: **Yes:**   Norhan S ElMongy

Reviewer #4: **Yes:**   Bernardo Spagnolo

**********

[NOTE: If reviewer comments were submitted as an attachment file, they will be attached to this email and accessible via the submission site. Please log into your account, locate the manuscript record, and check for the action link "View Attachments". If this link does not appear, there are no attachment files.]

To ensure your figures meet our technical requirements, please review our figure guidelines: https://journals.plos.org/plosone/s/figures

You may also use PLOS’s free figure tool, NAAS, to help you prepare publication quality figures: https://journals.plos.org/plosone/s/figures#loc-tools-for-figure-preparation.

NAAS will assess whether your figures meet our technical requirements by comparing each figure against our figure specifications.

Attachment Submitted filename: Reveiwers suggestions for this essay.doc

Attachment Submitted filename: Report_PONE-D-25-43663_R1_17.02.26 .pdf

# Author comment: Author response to Decision Letter 2

**DOI:** 10.1371/journal.pone.0351953.r004

17 Mar 2026

Response to Reviewers

Dear Editors and Reviewers,

We express our sincere gratitude to you and your colleagues for reviewing our submitted manuscript and providing valuable comments and suggestions. We highly appreciate these invaluable suggestions and believe they have played a crucial role in our research. We have carefully considered each of your suggestions, conducting thorough revisions to ensure the academic and methodological accuracy and credibility of our paper. Here, we sincerely respond to your review comments, hoping that our responses meet your expectations. Once again, we thank you for your valuable feedback and patience. Below are our responses to the specific comments:

1. Reviewer's Comment: “While the manuscript reports improvements in DSC and HD metrics, no statistical significance testing is provided to support these performance gains. Given that some improvements are relatively modest in magnitude, it would be important to demonstrate whether the reported differences are statistically significant across folds. Reporting mean ± standard deviation and including appropriate paired statistical tests (e.g., paired t-test or Wilcoxon signed-rank test) would strengthen the robustness of the conclusions.”

Response: We sincerely thank the reviewer for this rigorous and crucial suggestion. We completely agree that relying solely on average metrics is insufficient to prove the absolute superiority of the model, and that statistical significance testing is essential to confirm that the observed improvements are robust across different cross-validation folds. Following your valuable advice, we have comprehensively updated our quantitative evaluation:

1. We have revised the result tables (specifically the ablation study tables, e.g., Tables 3, 4, and 5) to report all metrics in the format of Mean ± Standard Deviation (SD) across the five-fold cross-validation. Furthermore, we conducted paired t-tests to compare the baseline models with our proposed GL-Net. The resulting p-values are now included in the tables (values in parentheses), where p<0.01 indicates high statistical significance. The results confirm that our performance gains, even those with smaller margins, are indeed statistically significant.

2. To formally document this statistical evaluation, we have added a dedicated paragraph to the “Dataset and evaluation metrics” section in the revised manuscript: “Furthermore, to rigorously validate the performance improvements of the proposed GL-Net, statistical significance testing was conducted. The quantitative segmentation results across the five-fold cross-validation are reported as Mean ± Standard Deviation (SD). A paired Student’s t-test was utilized to determine whether the performance differences between the baseline configurations (or comparative models) and the proposed GL-Net were statistically significant across the test folds. A p-value of <0.05 was considered statistically significant, and p<0.01 was considered highly significant.”

3. In addition to updating the tables with Mean ± SD and p-values, we have also expanded the text in the ‘Ablation experiments’ section to deeply analyze these statistical findings. Most notably, in the analysis of our proposed DiceBRD loss function (Table 5), we explicitly highlighted that while the overall volume overlap (DSC) showed moderate improvements, the reductions in boundary errors (Hausdorff Distance) were highly statistically significant (p≤0.01). This statistical evidence perfectly corroborates our methodological claim that the dynamic boundary weighting specifically and robustly refines fuzzy edge contours.

2. Reviewer's Comment: “Tables 1 and 2 compare GL-Net with several existing models. However, it is not clearly stated whether these models were re-implemented under identical preprocessing and cross-validation settings, or whether the results were directly cited from the original publications. This distinction is important for assessing fairness and reproducibility. Explicit clarification of the comparison protocol would improve methodological transparency.”

Response: We sincerely thank the reviewer for pointing out this important detail regarding methodological transparency. We completely agree that clarifying the evaluation protocol is crucial for fairness and reproducibility.

We would like to clarify that the quantitative results of the comparative models presented in Tables 1 and 2 were directly cited from their respective original publications, rather than being re-implemented under our exact preprocessing and cross-validation conditions. To ensure transparency, we have added explicit statements to the revised manuscript acknowledging this.

Specifically, we have updated the text in the "Comparative Experiments of GL-Net" section to read: "It is important to note that the quantitative results of the comparative models presented in these tables are directly cited from their respective original publications rather than re-implemented in our exact experimental environment. While this provides a comprehensive benchmark against current state-of-the-art methods, we acknowledge that minor variations in data splits (e.g., cross-validation folds) and preprocessing pipelines among these original studies may exist."

Additionally, we have added a clarifying note to the footer of both Table 1 and Table 2: "The performance metrics of the compared models are directly cited from their original publications."

3. Reviewer's Comment: “The mathematical formulation of the DiceBRD loss function requires clearer explanation. In particular, the relationship between the ROI-based region restriction and the combined Dice and weighted cross-entropy terms could be more explicitly described. Additionally, certain variable definitions appear repetitive or ambiguous. A more precise and structured presentation of the loss formulation would enhance technical clarity.”

Response: We sincerely thank the reviewer for this highly constructive and precise feedback. We agree that the mathematical formulation of the DiceBRD loss function in the original manuscript lacked sufficient rigor and contained typographical ambiguities.

In the revised manuscript, we have comprehensively restructured the presentation of the DiceBRD loss to ensure technical clarity:

1. We have corrected the typographical error where y_ic was ambiguously defined twice. It is now correctly defined as the ground truth one-hot encoding (y_ic∈{0,1}), while p_ic∈[0,1] is strictly defined as the predicted probability.

2. We replaced the vague terms N and voxels with standard set notations. We now use R to denote the dynamically extracted boundary region of interest, ∣R∣ for its cardinality (number of voxels), and V to denote the set of all voxels in the entire image volume.

3. We explicitly clarified the relationship between the two loss terms. The revised text now clearly states that the Dice loss term operates on the global domain V to capture overall topological structure and mitigate class imbalance, whereas the dynamic weighted cross-entropy term (L_BRD) is strictly restricted to the local boundary domain R (i∈R). This decoupling explains why the combination is highly effective at refining fuzzy edges without losing global context.

4. Reviewer's Comment: “The manuscript evaluates segmentation performance indirectly through downstream GBM classification using selected VASARI features. While the reported consistency between GL-Net-derived and ground-truth-derived features is encouraging, the manuscript could more clearly articulate how classification consistency serves as a direct validation of segmentation quality. A brief clarification of this methodological rationale would strengthen the interpretability of the clinical validation.”

Response: We sincerely thank the reviewer for this insightful comment. We completely agree that explicitly stating the methodological rationale strengthens the logical link between our downstream clinical task and the upstream segmentation quality.

While geometric evaluation metrics (such as DSC and HD) are standard for assessing pixel-level spatial overlap, they do not necessarily reflect the clinical utility of the segmentation. In clinical practice, the key requirement of tumor segmentation is to accurately capture the structural and compositional features (e.g., the proportions of necrosis, enhancing, and non-enhancing subregions) that directly drive diagnostic decisions. Because the extraction of VASARI features heavily relies on the precise morphological boundaries and volume ratios of these subregions, achieving highly consistent downstream classification performance (and identical feature weight distributions) using GL-Net-generated masks compared to expert manual annotations (ground truth) directly proves that GL-Net accurately preserves clinically relevant morphological variations. This translates geometric accuracy into genuine clinical reliability.

To make this rationale clear to the readers, we have added an explanatory paragraph at the beginning of the "VASARI-based Feature Analysis" subsection in the revised manuscript:

"While geometric metrics such as DSC and HD provide quantitative measures of spatial overlap and boundary distances, they do not fully capture the clinical utility of a segmentation model. In clinical workflows, the ultimate goal of tumor delineation is to extract reliable morphological and compositional metrics—such as the proportions of necrosis, enhancing, and non-enhancing tumor regions—that directly inform diagnosis and treatment planning. Therefore, downstream classification consistency serves as a crucial clinical validation of segmentation quality. If the segmentation masks generated by GL-Net yield VASARI features and subsequent GBM diagnostic performance that are statistically indistinguishable from those derived from expert manual annotations (ground truth), it directly demonstrates that GL-Net successfully preserves the clinically critical morphological variations and subregion proportions required for accurate diagnosis, thereby bridging the gap between pixel-level accuracy and clinical reliability."

5. Reviewer's Comment: “Although the manuscript discusses clinical integration and workflow applicability, no external validation on independent real-world cohorts is provided. The current evaluation is limited to curated BraTS datasets. The claims regarding clinical applicability should be interpreted in light of this limitation, and a brief acknowledgment of the need for further prospective validation may improve balance.”

Response: We sincerely thank the reviewer for highlighting this critical limitation. We completely agree that performing well on curated public datasets, such as BraTS, does not automatically guarantee equivalent robustness in noisy, uncurated, real-world clinical environments. Our previous claims regarding clinical applicability were perhaps too optimistic without emphasizing this necessary caveat.

To provide a more balanced and scientifically rigorous perspective, we have tempered our claims regarding direct clinical applicability and explicitly acknowledged the lack of independent external validation as a primary limitation of our current study.

Specifically, we have substantially revised the final paragraph of the Conclusion section to read:

"While GL-Net demonstrates strong segmentation accuracy and reliable downstream feature extraction, claims regarding its direct clinical applicability must be interpreted with caution. A primary limitation of the current study is the reliance on the highly curated BraTS datasets. Although multi-institutional, these standardized datasets may not fully capture the extreme heterogeneity, unpredictable artifacts, and diverse scanning protocols encountered in independent, real-world clinical cohorts. The absence of external validation on an independent real-world dataset means the model's operational robustness in everyday clinical practice remains to be definitively proven. Therefore, extensive external validation on independent clinical cohorts and subsequent prospective trials are essential next steps to rigorously evaluate the model's true generalizability..."

6. Reviewer's Comment: “For full reproducibility, additional implementation details would be helpful. The manuscript does not specify the computational environment (e.g., GPU type), training time, inference time, or model parameter size. Including these details would improve transparency and practical reproducibility.”

Response: We sincerely thank the reviewer for this highly practical and constructive suggestion. We completely agree that detailing the computational environment, model size, and time efficiency is essential for ensuring full transparency and reproducibility, as well as for evaluating the feasibility of clinical deployment.

In response to your valuable feedback, we have updated the "Experimental Setup and Preprocessing" section of the revised manuscript to include all the requested implementation details. Specifically, we have added the following information:

"All experiments were conducted on an Ubuntu operating system equipped with an Intel(R) Xeon(R) CPU E5-2678 v3 @ 2.50GHz, 128 GB of system RAM, and dual NVIDIA RTX 3090 Ti GPUs (24 GB VRAM each) …"

7. Reviewer's Comment: “The manuscript refers to the ‘BraTS2021 test set,’ while also stating that five-fold cross-validation was performed. Since official test labels are not publicly available in BraTS challenges, the terminology should be clarified to avoid confusion regarding evaluation protocol.”

Response: We sincerely thank the reviewer for pointing out this terminological ambiguity. To avoid any confusion, we would like to clarify that because the official validation and test set ground truths are not publicly available, all evaluations in our study were conducted exclusively on the official BraTS training datasets. The "test sets" mentioned in our original manuscript were, in fact, the internal hold-out test folds generated during our five-fold cross-validation process on the official training data.

To ensure absolute clarity regarding our evaluation protocol, we have thoroughly revised the terminology throughout the manuscript:

1. In the "Dataset and evaluation metrics" section, we added an explicit statement: "Since the ground truth labels for the official validation and test sets of BraTS2019 and BraTS2021 are withheld by the challenge organizers and are not publicly available, all models in this study were trained and evaluated exclusively on the official training datasets. To ensure a robust and fair evaluation, a five-fold cross-validation strategy was performed. Therefore, any reference to "test set" or "test results" in this paper specifically denotes the internal hold-out test folds partitioned during this cross-validation process, rather than the official unseen BraTS test sets."

2. In the "Comparative Experiments of GL-Net" section and table captions (e.g., Table 5), we replaced the misleading phrase "BraTS test set" with "internal hold-out test sets" or "internal test set".

Once again, we would like to express our deepest appreciation to the Editors and the Reviewers for their time, expertise, and highly constructive feedback. By addressing your insightful comments—specifically by incorporating rigorous statistical significance testing, clarifying mathematical formulations, providing detailed implementation metrics, standardizing our evaluation terminology, and offering a more balanced discussion on clinical applicability—we believe the methodological transparency, scientific rigor, and overall quality of our manuscript have been substantially elevated.

We hope that these comprehensive revisions satisfactorily address all your concerns and that the revised manuscript is now deemed suitable for publication in your journal. We remain fully available should you require any further clarifications or modifications.

Sincerely,

Huimin Lu

Attachment Submitted filename: Response_to_Reviewers_auresp_2.docx

# Editor report: Decision Letter 2

**DOI:** 10.1371/journal.pone.0351953.r005

**Authors:** Taikyeong Jeong

9 Apr 2026

PONE-D-25-43663R2

GL-Net: A Knowledge-Guided Gaussian-Gated and Layered Refinement Network for 3D MRI Segmentation of Brain Gliomas

PLOS One

Dear Dr. Lu,

Thank you for submitting your manuscript to PLOS ONE. After careful consideration, we feel that it has merit but does not fully meet PLOS ONE’s publication criteria as it currently stands. Therefore, we invite you to submit a revised version of the manuscript that addresses the points raised during the review process.

Please submit your revised manuscript by May 24 2026 11:59PM. If you will need more time than this to complete your revisions, please reply to this message or contact the journal office at plosone@plos.org. When you're ready to submit your revision, log on to https://www.editorialmanager.com/pone/ and select the 'Submissions Needing Revision' folder to locate your manuscript file.

Please include the following items when submitting your revised manuscript:

- A letter that responds to each point raised by the academic editor and reviewer(s). You should upload this letter as a separate file labeled 'Response to Reviewers'.
- A marked-up copy of your manuscript that highlights changes made to the original version. You should upload this as a separate file labeled 'Revised Manuscript with Track Changes'.
- An unmarked version of your revised paper without tracked changes. You should upload this as a separate file labeled 'Manuscript'.

If you would like to make changes to your financial disclosure, please include your updated statement in your cover letter. Guidelines for resubmitting your figure files are available below the reviewer comments at the end of this letter.

If applicable, we recommend that you deposit your laboratory protocols in protocols.io to enhance the reproducibility of your results. Protocols.io assigns your protocol its own identifier (DOI) so that it can be cited independently in the future. For instructions see: https://journals.plos.org/plosone/s/submission-guidelines#loc-laboratory-protocols. Additionally, PLOS ONE offers an option for publishing peer-reviewed Lab Protocol articles, which describe protocols hosted on protocols.io. Read more information on sharing protocols at https://plos.org/protocols?utm_medium=editorial-email&utm_source=authorletters&utm_campaign=protocols.

As the corresponding author, your ORCID iD is verified in the submission system and will appear in the published article. PLOS supports the use of ORCID, and we encourage all coauthors to register for an ORCID iD and use it as well. Please encourage your coauthors to verify their ORCID iD within the submission system before final acceptance, as unverified ORCID iDs will not appear in the published article. *Only*   the individual author can complete the verification step; PLOS staff *cannot*   verify ORCID iDs on behalf of authors.

We look forward to receiving your revised manuscript.

Kind regards,

Taikyeong Ted Jeong, Ph.D.

Academic Editor

PLOS One

Journal Requirements:

If the reviewer comments include a recommendation to cite specific previously published works, please review and evaluate these publications to determine whether they are relevant and should be cited. There is no requirement to cite these works unless the editor has indicated otherwise.

Please review your reference list to ensure that it is complete and correct. If you have cited papers that have been retracted, please include the rationale for doing so in the manuscript text, or remove these references and replace them with relevant current references. Any changes to the reference list should be mentioned in the rebuttal letter that accompanies your revised manuscript. If you need to cite a retracted article, indicate the article’s retracted status in the References list and also include a citation and full reference for the retraction notice.

Additional Editor Comments:

At this point, the authors have provided a detailed and well-structured response to the reviewer comments.

The rebuttal is generally thorough, respectful, and demonstrates a clear effort to revise the manuscript accordingly. Most concerns raised in the previous round have been addressed to a reasonable extent.

However, while the revision has improved the manuscript, several issues remain insufficiently resolved or would benefit from further clarification.

The authors have incorporated mean ± standard deviation and conducted paired t-tests across cross-validation folds, which is a welcome improvement.

However, some concerns remain:

The manuscript does not clarify whether the assumptions of the paired t-test (e.g., normality) were verified.

Given the limited number of folds (n=5), the robustness of statistical testing may still be questioned.

The authors should provide additional justification for the choice of statistical test and, if possible, include complementary non-parametric testing or normality verification.

The authors clarify that the results of comparative models were directly cited from the literature rather than re-implemented under identical experimental conditions.

While this improves transparency, it raises concerns regarding fairness:

Differences in preprocessing, training protocols, and data splits may significantly affect performance.

Therefore, the comparison may not be strictly controlled.

The authors should more explicitly acknowledge this limitation and moderate claims of superiority. Ideally, re-implementation under a unified framework would strengthen the validity of comparisons, though this may be left as future work.

The authors have improved the clarity of variable definitions and provided a more structured explanation of the loss components.

However: the final mathematical formulation of the combined loss function is still not explicitly presented in a concise equation.

The relationship between the global Dice loss and the local boundary-restricted term would benefit from a clearer formal expression.

Include an explicit final loss equation and ensure all symbols are consistently defined.

The authors provide a more detailed explanation of using VASARI-based classification consistency as a proxy for segmentation quality.

This clarification is helpful. However: the argument remains somewhat overstated.

The claim that downstream consistency “directly proves” segmentation quality is too strong.

Rephrase this argument to reflect that downstream consistency provides supporting or indirect evidence, rather than definitive proof.

The authors have appropriately acknowledged the limitation regarding the lack of external validation and have moderated their claims.

Well, this is a strong improvement, and the revised discussion is now more balanced and scientifically appropriate.

Also, the addition of hardware specifications is appreciated.

However, important details are still missing: Model parameter size, Training time, Inference time

Include these details to ensure full reproducibility and to allow readers to assess computational feasibility.

The authors have clarified the misuse of the term “test set” and corrected the terminology throughout the manuscript.

The authors have made substantial improvements in response to the reviewer comments, particularly in terms of statistical analysis, transparency, and clarity.

However, several points still require minor but important revisions, particularly regarding:

Statistical rigor, Fairness of comparisons , Mathematical clarity, Reproducibility details

[Note: HTML markup is below. Please do not edit.]

[NOTE: If reviewer comments were submitted as an attachment file, they will be attached to this email and accessible via the submission site. Please log into your account, locate the manuscript record, and check for the action link "View Attachments". If this link does not appear, there are no attachment files.]

To ensure your figures meet our technical requirements, please review our figure guidelines: https://journals.plos.org/plosone/s/figures

You may also use PLOS’s free figure tool, NAAS, to help you prepare publication quality figures: https://journals.plos.org/plosone/s/figures#loc-tools-for-figure-preparation.

NAAS will assess whether your figures meet our technical requirements by comparing each figure against our figure specifications.

# Author comment: Author response to Decision Letter 3

**DOI:** 10.1371/journal.pone.0351953.r006

20 Apr 2026

Response to Reviewers

Dear Editors and Reviewers,

We express our sincere gratitude to you and your colleagues for reviewing our submitted manuscript and providing valuable comments and suggestions. We highly appreciate these invaluable suggestions and believe they have played a crucial role in our research. We have carefully considered each of your suggestions, conducting thorough revisions to ensure the academic and methodological accuracy and credibility of our paper. Here, we sincerely respond to your review comments, hoping that our responses meet your expectations. Once again, we thank you for your valuable feedback and patience. Below are our responses to the specific comments:

1. Reviewer's Comment: “The authors have incorporated mean ± standard deviation and conducted paired t-tests across cross-validation folds, which is a welcome improvement. However, some concerns remain: The manuscript does not clarify whether the assumptions of the paired t-test (e.g., normality) were verified. Given the limited number of folds (n=5), the robustness of statistical testing may still be questioned.”

Response: We sincerely thank the reviewer for this insightful and constructive comment. We apologize for the lack of clarity regarding our statistical testing protocol and the rationale behind our cross-validation strategy. To address your concerns, we have clarified our methodology below and updated the manuscript accordingly.

1. We completely agree that strictly verifying the normality assumption is the best practice. Retrospectively, we conducted the Shapiro-Wilk test on the case-level differences. While most regions approximated normal distributions, a few exhibited slight skewness. To ensure absolute statistical rigor, we additionally performed the non-parametric Wilcoxon signed-rank test, which does not assume a normal distribution. The resulting p-values from the Wilcoxon test remain highly significant (p<0.01), perfectly consistent with the conclusions drawn from our original t-tests. We have updated the revised manuscript to explicitly describe this robust statistical procedure.

2. We deeply appreciate the reviewer’s concern regarding the number of folds. The choice of 5-fold cross-validation was a deliberate decision based on the bias-variance tradeoff. In our datasets (e.g., N=335 for BraTS 2019), increasing the number of folds (e.g., to 10-fold) would reduce the size of each validation set to only about 33 samples. Smaller validation sets are prone to high variance, meaning the performance metrics become overly sensitive to individual heterogeneous samples or noise, potentially leading to unreliable estimates of model generalization. A 5-fold CV ensures a more substantial validation set (n=67 per fold), providing a much more stable and representative estimate of the model. Furthermore, calculating 3D MRI configurations is computationally intensive; the 5-fold strategy optimally balances statistical stability and computational feasibility.

2. Reviewer's Comment: “The authors clarify that the results of comparative models were directly cited from the literature rather than re-implemented under identical experimental conditions. While this improves transparency, it raises concerns regarding fairness: Differences in preprocessing, training protocols, and data splits may significantly affect performance. Therefore, the comparison may not be strictly controlled. The authors should more explicitly acknowledge this limitation and moderate claims of superiority. Ideally, re-implementation under a unified framework would strengthen the validity of comparisons, though this may be left as future work.”

Response: We sincerely thank the reviewer for pointing out this critical caveat. We completely agree that comparing our results directly with those cited from the literature—without strictly controlling for preprocessing, exact cross-validation splits, and hardware/software environments—introduces potential biases and compromises the strict fairness of the comparison. we have made the following specific revisions to the manuscript:

1. We have significantly expanded the discussion in the “Comparative Experiments of GL-Net” section to explicitly warn readers that differences in data splits and pipelines may impact the performance metrics, and thus the comparisons should be interpreted as contextual benchmarks rather than strictly controlled head-to-head evaluations.

2. We have systematically reviewed the manuscript (especially the Abstract, Results, and Conclusion sections) and toned down definitive claims of “absolute superiority” (e.g., changing phrases like “outperforming several state-of-the-art algorithms” to “demonstrating highly competitive performance relative to reported benchmarks”).

3. As suggested, we have added a dedicated sentence in the “Conclusion” section stating that re-implementing these baseline models within a unified framework (e.g., standardized data splits and preprocessing pipelines) remains a crucial direction for our future work to establish more reliable comparisons.

3. Reviewer's Comment: “The authors have improved the clarity of variable definitions and provided a more structured explanation of the loss components. However: the final mathematical formulation of the combined loss function is still not explicitly presented in a concise equation. The relationship between the global Dice loss and the local boundary-restricted term would benefit from a clearer formal expression. Include an explicit final loss equation and ensure all symbols are consistently defined.”

Response: To address this issue, we have thoroughly revised the “Dynamic Weighted Loss Function for Edge Region Voxels” section. We have now decomposed the complex equation into three highly concise and explicit mathematical formulas:

1. The global Dice loss (L_Dice).

2. The boundary-restricted dynamic weighted cross-entropy loss (L_BRD).

3. The final combined loss equation expressed as a weighted sum (L_DiceBRD=L_Dice+λL_BRD), which explicitly illustrates the relationship between the global and local terms.

Furthermore, immediately following these equations, we have provided a unified, bulleted list to define every mathematical symbol (V,R,∣R∣,C,y_ic,p_ic,w_i,ε,λ) explicitly and consistently, leaving no room for ambiguity. We believe these modifications greatly enhance the mathematical readability and exactness of our proposed method. Please see the revised manuscript for these specific changes.

4. Reviewer's Comment: “The authors provide a more detailed explanation of using VASARI-based classification consistency as a proxy for segmentation quality. This clarification is helpful. However: the argument remains somewhat overstated. The claim that downstream consistency “directly proves” segmentation quality is too strong. Rephrase this argument to reflect that downstream consistency provides supporting or indirect evidence, rather than definitive proof.”

Response: We sincerely appreciate the reviewer’s careful reading and valid critique regarding the precision of our scientific claims. We completely agree that claiming downstream classification consistency “directly demonstrates” segmentation quality is overly absolute. While downstream task performance highlights the clinical utility of the generated masks, it is ultimately an indirect measure and should be properly framed as complementary evidence alongside geometric metrics like DSC and HD, rather than definitive proof of pixel-perfect segmentation. In accordance with your excellent suggestion, we have systematically reviewed the manuscript and meticulously toned down these claims, particularly in the “Abstract”, “VASARI-based Feature Analysis”, and “Conclusion” sections. We have rephrased the relevant sentences to explicitly state that downstream consistency “provides valuable complementary and indirect evidence” of the model’s reliability and clinical utility. We believe these revisions significantly enhance the scientific rigor and objectivity of our manuscript.

5. Reviewer's Comment: “The authors have appropriately acknowledged the limitation regarding the lack of external validation and have moderated their claims. Well, this is a strong improvement, and the revised discussion is now more balanced and scientifically appropriate. Also, the addition of hardware specifications is appreciated. However, important details are still missing: Model parameter size, Training time, Inference time. Include these details to ensure full reproducibility and to allow readers to assess computational feasibility.”

Response: We sincerely thank the reviewer for the highly encouraging feedback and for recognizing the improvements in our revised manuscript. We also appreciate you emphasizing the importance of computational details.

We apologize if these specific metrics were not prominent enough in our previous revision, which might have caused them to be overlooked. We are pleased to clarify that we have indeed included these exact details at the end of the “Experimental Setup and Preprocessing” section. To ensure absolute clarity, we summarize them here:

1. Model Parameter Size: The proposed GL-Net has a total parameter count of approximately 35.2 M;

2. Training Time: Under our hardware configuration (dual NVIDIA RTX 3090 Ti GPUs), the total training time for 100 epochs was approximately 14 hours.

3. Inference Time: During the testing phase, the average inference time per patient volume (a full 3D MRI scan) was strictly around 4.2 seconds (approx. 27 ms per slice).

We hope that these comprehensive revisions satisfactorily address all your concerns and that the revised manuscript is now deemed suitable for publication in your journal

Sincerely,

Huimin Lu

Attachment Submitted filename: Response_to_Reviewers_auresp_3.docx

# Editor report: Decision Letter 3

**DOI:** 10.1371/journal.pone.0351953.r007

**Authors:** Taikyeong Jeong

4 May 2026

PONE-D-25-43663R3

GL-Net: A Knowledge-Guided Gaussian-Gated and Layered Refinement Network for 3D MRI Segmentation of Brain Gliomas

PLOS One

Dear Dr. Lu,

Thank you for submitting your manuscript to PLOS ONE. After careful consideration, we feel that it has merit but does not fully meet PLOS ONE’s publication criteria as it currently stands. Therefore, we invite you to submit a revised version of the manuscript that addresses the points raised during the review process.

==============================

This paper presents a knowledge-based deep learning framework (GL-Net) for glioma segmentation through validation based on the BraTS dataset and VASARI. This study is methodologically valid, and the experimental design (including resection and statistical analysis) is suitable for publication in a journal. However, while suitable for publication, several points regarding interpretation and reporting must be modified.

(1) The claim of superiority over existing methods should be softened, as comparisons were not made in a strictly controlled environment. Please revise it to reflect competitive performance. The author is using very dangerous wording. "Comparison is not strictly controlled..." is incomprehensible to an experimenter.

(2) Clinical claims should be softened. In the absence of external validation, expressions such as "clinical utility" should be revised to indicate potential clinical relevance.

(3) p-values reported as "0.00" should be corrected to a standard format (e.g., p < 0.01)

==============================

Please submit your revised manuscript by Jun 18 2026 11:59PM. If you will need more time than this to complete your revisions, please reply to this message or contact the journal office at plosone@plos.org. When you're ready to submit your revision, log on to https://www.editorialmanager.com/pone/ and select the 'Submissions Needing Revision' folder to locate your manuscript file.

Please include the following items when submitting your revised manuscript:

- A letter that responds to each point raised by the academic editor and reviewer(s). You should upload this letter as a separate file labeled 'Response to Reviewers'.
- A marked-up copy of your manuscript that highlights changes made to the original version. You should upload this as a separate file labeled 'Revised Manuscript with Track Changes'.
- An unmarked version of your revised paper without tracked changes. You should upload this as a separate file labeled 'Manuscript'.

If you would like to make changes to your financial disclosure, please include your updated statement in your cover letter. Guidelines for resubmitting your figure files are available below the reviewer comments at the end of this letter.

If applicable, we recommend that you deposit your laboratory protocols in protocols.io to enhance the reproducibility of your results. Protocols.io assigns your protocol its own identifier (DOI) so that it can be cited independently in the future. For instructions see: https://journals.plos.org/plosone/s/submission-guidelines#loc-laboratory-protocols. Additionally, PLOS ONE offers an option for publishing peer-reviewed Lab Protocol articles, which describe protocols hosted on protocols.io. Read more information on sharing protocols at https://plos.org/protocols?utm_medium=editorial-email&utm_source=authorletters&utm_campaign=protocols.

As the corresponding author, your ORCID iD is verified in the submission system and will appear in the published article. PLOS supports the use of ORCID, and we encourage all coauthors to register for an ORCID iD and use it as well. Please encourage your coauthors to verify their ORCID iD within the submission system before final acceptance, as unverified ORCID iDs will not appear in the published article. *Only*   the individual author can complete the verification step; PLOS staff *cannot*   verify ORCID iDs on behalf of authors.

We look forward to receiving your revised manuscript.

Kind regards,

Taikyeong Ted Jeong, Ph.D.

Academic Editor

PLOS One

Journal Requirements:

If the reviewer comments include a recommendation to cite specific previously published works, please review and evaluate these publications to determine whether they are relevant and should be cited. There is no requirement to cite these works unless the editor has indicated otherwise.

Please review your reference list to ensure that it is complete and correct. If you have cited papers that have been retracted, please include the rationale for doing so in the manuscript text, or remove these references and replace them with relevant current references. Any changes to the reference list should be mentioned in the rebuttal letter that accompanies your revised manuscript. If you need to cite a retracted article, indicate the article’s retracted status in the References list and also include a citation and full reference for the retraction notice.

Additional Editor Comments :

This paper presents a knowledge-based deep learning framework (GL-Net) for glioma segmentation through validation based on the BraTS dataset and VASARI. This study is methodologically valid, and the experimental design (including resection and statistical analysis) is suitable for publication in a journal. However, while suitable for publication, several points regarding interpretation and reporting must be modified.

(1) The claim of superiority over existing methods should be softened, as comparisons were not made in a strictly controlled environment. Please revise it to reflect competitive performance. The author is using very dangerous wording. "Comparison is not strictly controlled..." is incomprehensible to an experimenter.

(2) Clinical claims should be softened. In the absence of external validation, expressions such as "clinical utility" should be revised to indicate potential clinical relevance.

(3) p-values reported as "0.00" should be corrected to a standard format (e.g., p < 0.01).

[Note: HTML markup is below. Please do not edit.]

[NOTE: If reviewer comments were submitted as an attachment file, they will be attached to this email and accessible via the submission site. Please log into your account, locate the manuscript record, and check for the action link "View Attachments". If this link does not appear, there are no attachment files.]

To ensure your figures meet our technical requirements, please review our figure guidelines: https://journals.plos.org/plosone/s/figures

You may also use PLOS’s free figure tool, NAAS, to help you prepare publication quality figures: https://journals.plos.org/plosone/s/figures#loc-tools-for-figure-preparation.

NAAS will assess whether your figures meet our technical requirements by comparing each figure against our figure specifications.

# Author comment: Author response to Decision Letter 4

**DOI:** 10.1371/journal.pone.0351953.r008

8 May 2026

Response to Reviewers

Dear Editors and Reviewers,

We express our sincere gratitude to you and your colleagues for reviewing our submitted manuscript and providing valuable comments and suggestions. We highly appreciate these invaluable suggestions and believe they have played a crucial role in our research. We have carefully considered each of your suggestions, conducting thorough revisions to ensure the academic and methodological accuracy and credibility of our paper. Here, we sincerely respond to your review comments, hoping that our responses meet your expectations. Once again, we thank you for your valuable feedback and patience. Below are our responses to the specific comments:

1. Reviewer's Comment: “The claim of superiority over existing methods should be softened, as comparisons were not made in a strictly controlled environment. Please revise it to reflect competitive performance. The author is using very dangerous wording. "Comparison is not strictly controlled..." is incomprehensible to an experimenter.”

Response: We sincerely thank the reviewer for this critical and highly constructive feedback. We completely agree that claiming absolute superiority is scientifically inappropriate when comparing our results with metrics directly cited from other publications, given the inevitable differences in data splits, preprocessing pipelines, and evaluation frameworks. We also deeply appreciate the reviewer pointing out the inappropriate and potentially misleading phrase "Comparison is not strictly controlled". To address this, we have thoroughly revised the manuscript. Specifically, we have:

1. Removed all variations of the phrase "not strictly controlled" and replaced them with standard academic terminology (e.g., "indirect comparison," "different evaluation frameworks").

2. Softened words like "superior," "outperforming," and "best" to "highly competitive," "comparable to state-of-the-art," and "demonstrates robust performance" throughout the Abstract, Results, and Conclusion sections.

3. 3. Clarified that our results show the model's strong potential and competitiveness, while explicitly acknowledging that a definitive head-to-head evaluation would require re-implementing all models within a unified framework (such as nnU-Net).

2. Reviewer's Comment: “Clinical claims should be softened. In the absence of external validation, expressions such as "clinical utility" should be revised to indicate potential clinical relevance.”

Response: We sincerely thank the reviewer for pointing this out. We fully agree that without validation on an independent, external clinical dataset, claiming "clinical utility" or "direct clinical applicability" is scientifically premature and overstates the current developmental stage of our model.

To ensure scientific rigor and adhere to standard reporting practices in medical image analysis, we have thoroughly reviewed the manuscript and softened all relevant clinical claims. Specifically, we have replaced terms such as "clinical utility" and "practical utility" with more objective and cautious expressions, including "potential clinical relevance," "translational potential," and "clinical potential." Furthermore, we have explicitly highlighted the critical need for future external validation on independent cohorts before any true clinical utility can be established. We believe these revisions accurately reflect the current limitations and the true scope of our work.

3. Reviewer's Comment: “p-values reported as "0.00" should be corrected to a standard format (e.g., p < 0.01).”

Response: We sincerely thank the reviewer for pointing out this formatting issue. We completely agree that reporting a p-value exactly as "0.00" is statistically inaccurate. Following your suggestion, we have carefully reviewed the entire manuscript, including all relevant texts and tables (Tables 3, 4, and 5). We have corrected all instances of "0.00" to the standard format "< 0.01". Accordingly, we have also updated the table footnotes to reflect these changes. We appreciate your rigorous attention to detail, which has helped us improve the statistical reporting standard of our paper.

We hope that these comprehensive revisions satisfactorily address all your concerns and that the revised manuscript is now deemed suitable for publication in your journal

Sincerely,

Huimin Lu

Attachment Submitted filename: Response_to_Reviewers_auresp_4.docx

# Editor report: Decision Letter 4

**DOI:** 10.1371/journal.pone.0351953.r009

**Authors:** Taikyeong Jeong

3 Jun 2026

GL-Net: A Knowledge-Guided Gaussian-Gated and Layered Refinement Network for 3D MRI Segmentation of Brain Gliomas

PONE-D-25-43663R4

Dear Dr. Lu,

We’re pleased to inform you that your manuscript has been judged scientifically suitable for publication and will be formally accepted for publication once it meets all outstanding technical requirements.

Within one week, you’ll receive an e-mail detailing the required amendments. When these have been addressed, you’ll receive a formal acceptance letter and your manuscript will be scheduled for publication.

An invoice will be generated when your article is formally accepted. Please note, if your institution has a publishing partnership with PLOS and your article meets the relevant criteria, all or part of your publication costs will be covered. Please make sure your user information is up-to-date by logging into Editorial Manager at Editorial Manager® and clicking the ‘Update My Information' link at the top of the page. For questions related to billing, please contact billing support.

If your institution or institutions have a press office, please notify them about your upcoming paper to help maximize its impact. If they’ll be preparing press materials, please inform our press team as soon as possible -- no later than 48 hours after receiving the formal acceptance. Your manuscript will remain under strict press embargo until 2 pm Eastern Time on the date of publication. For more information, please contact onepress@plos.org.

Kind regards,

Taikyeong Ted Jeong, Ph.D.

Academic Editor

PLOS One

Additional Editor Comments (optional):

As an academic editor, I appreciate the author's thoughtful response and hope for its publication in this journal.

Reviewers' comments:

# Editor report: Acceptance letter

**DOI:** 10.1371/journal.pone.0351953.r010

**Authors:** Taikyeong Jeong

PONE-D-25-43663R4

PLOS One

Dear Dr. Lu,

I'm pleased to inform you that your manuscript has been deemed suitable for publication in PLOS One. Congratulations! Your manuscript is now being handed over to our production team.

At this stage, our production department will prepare your paper for publication. This includes ensuring the following:

* All references, tables, and figures are properly cited

* All relevant supporting information is included in the manuscript submission,

* There are no issues that prevent the paper from being properly typeset

You will receive further instructions from the production team, including instructions on how to review your proof when it is ready. Please keep in mind that we are working through a large volume of accepted articles, so please give us a few days to review your paper and let you know the next and final steps.

Lastly, if your institution or institutions have a press office, please let them know about your upcoming paper now to help maximize its impact. If they'll be preparing press materials, please inform our press team within the next 48 hours. Your manuscript will remain under strict press embargo until 2 pm Eastern Time on the date of publication. For more information, please contact onepress@plos.org.

You will receive an invoice from PLOS for your publication fee after your manuscript has reached the completed accept phase. If you receive an email requesting payment before acceptance or for any other service, this may be a phishing scheme. Learn how to identify phishing emails and protect your accounts at https://explore.plos.org/phishing.

If we can help with anything else, please email us at customercare@plos.org.

Thank you for submitting your work to PLOS ONE and supporting open access.

Kind regards,

PLOS ONE Editorial Office Staff

on behalf of

Professor Taikyeong Ted Jeong

Academic Editor

PLOS One
